# Chimeric *Plasmodium falciparum* parasites expressing *Plasmodium vivax* circumsporozoite protein fail to produce salivary gland sporozoites

**DOI:** 10.1186/s12936-018-2431-1

**Published:** 2018-08-09

**Authors:** Catherin Marin-Mogollon, Fiona J. A. van Pul, Shinya Miyazaki, Takashi Imai, Jai Ramesar, Ahmed M. Salman, Beatrice M. F. Winkel, Ahmad Syibli Othman, Hans Kroeze, Severine Chevalley-Maurel, Arturo Reyes-Sandoval, Meta Roestenberg, Blandine Franke-Fayard, Chris J. Janse, Shahid M. Khan

**Affiliations:** 1 Department of Parasitology, Leiden University Medical Center, Albinusdreef 2, 2333 ZA Leiden, The Netherlands; 2 The Jenner Institute, Nuffield Department of Medicine, University of Oxford, The Henry Welcome Building for Molecular Physiology, Roosevelt Drive, Oxford, OX3 7BN UK; 3 Faculty of Health Sciences, Universiti Sultan Zainal Abidin, Terengganu, Malaysia; 4 Department of Infectious Diseases and Host Defense, Gunma University Graduate School of Medicine, Maebashi, Gunma 371-8510 Japan

**Keywords:** Malaria, *P. falciparum*, *P. vivax*, Circumsporozoite protein, CSP, Gene complementation

## Abstract

**Background:**

Rodent malaria parasites where the gene encoding circumsporozoite protein (CSP) has been replaced with *csp* genes from the human malaria parasites, *Plasmodium falciparum* or *Plasmodium vivax*, are used as pre-clinical tools to evaluate CSP vaccines in vivo. These chimeric rodent parasites produce sporozoites in *Anopheles stephensi* mosquitoes that are capable of infecting rodent and human hepatocytes. The availability of chimeric *P. falciparum* parasites where the *pfcsp* gene has been replaced by the *pvcsp* would open up possibilities to test *P. vivax* CSP vaccines in small scale clinical trials using controlled human malaria infection studies.

**Methods:**

Using CRISPR/Cas9 gene editing two chimeric *P. falciparum* parasites, were generated, where the *pfcsp* gene has been replaced by either one of the two major *pvcsp* alleles, VK210 or VK247. In addition, a *P. falciparum* parasite line that lacks CSP expression was also generated. These parasite lines have been analysed for sporozoite production in *An. stephensi* mosquitoes.

**Results:**

The two chimeric Pf-PvCSP lines exhibit normal asexual and sexual blood stage development in vitro and produce sporozoite-containing oocysts in *An. stephensi* mosquitoes. Expression of the corresponding PvCSP was confirmed in oocyst-derived Pf-PvCSP sporozoites. However, most oocysts degenerate before sporozoite formation and sporozoites were not found in either the mosquito haemocoel or salivary glands. Unlike the chimeric Pf-PvCSP parasites, oocysts of *P. falciparum* parasites lacking CSP expression do not produce sporozoites.

**Conclusions:**

Chimeric *P. falciparum* parasites expressing *P. vivax* circumsporozoite protein fail to produce salivary gland sporozoites. Combined, these studies show that while PvCSP can partially complement the function of PfCSP, species-specific features of CSP govern full sporozoite maturation and development in the two human malaria parasites.

**Electronic supplementary material:**

The online version of this article (10.1186/s12936-018-2431-1) contains supplementary material, which is available to authorized users.

## Background

*Plasmodium* sporozoites enter the blood stream through the bite of an infectious mosquito, after which they quickly migrate to the liver and invade hepatocytes. After multiplication within hepatocytes, merozoites are formed and released into the blood stream where they invade erythrocytes. Proteins of the pre-erythrocytic life cycle stages, sporozoites and liver stages, are attractive vaccine targets and are the principal components of leading malaria vaccines against the human parasites *Plasmodium falciparum* and *Plasmodium vivax* [1–4]. The target antigen of the most advanced *P. falciparum* malaria vaccine (RTS,S) is the circumsporozoite protein (CSP), the major sporozoite surface protein [5, 6] and is also an important vaccine target for *P. vivax* [7, 8]. CSP plays a critical role both in sporozoite formation and in sporozoite invasion of mosquito salivary glands and liver cells of the host [9–12]. So far, pre-erythrocytic subunit malaria vaccines, including RTS,S, have shown low to modest protective efficacy, both in the clinic and in field studies [13–16]. Efforts to increase the protective efficacy of malaria vaccines is focussed on identifying novel antigens, combining multiple antigens in a vaccine and by improving the delivery and immunogenicity of these antigens by using a variety of novel immunization platforms.

Testing the next generation of *P. falciparum* vaccines and vaccine formulations is greatly aided by the ability to vaccinate individuals and then examine vaccine efficacy by infecting them with malaria-parasites in so-called controlled human malaria infections (CHMI) [17–20]. CHMI studies have increased the speed of vaccine evaluation by using well-controlled early-phase proof-of-concept clinical studies. Such studies facilitate the down-selection of vaccine candidates and identifying those most suitable for further evaluation in more expensive and difficult phase 2 and 3 trials in areas where malaria is endemic.

Although recently CHMI has also been developed for *P. vivax* [21] and has been applied to assess pre-erythrocytic vaccine candidates [22, 23], the use of *P. vivax* CHMI to rapidly screen different *P. vivax* vaccines is limited because of the lack of methods to continuously propagate *P. vivax* blood stages in culture and to produce gametocytes in vitro that can be used to infect mosquitoes to produce sporozoites for challenge infections [21]. Therefore, *P. vivax* CHMI is dependent on sporozoites that have been obtained from mosquitoes fed on infected patients [21]. Moreover, *P. vivax* sporozoites can produce hypnozoites, dormant forms that can persist in the liver for prolonged periods, which requires safe and effective means to clear these forms from the liver in CHMI studies [21, 24].

In preclinical evaluation of vaccines, chimeric rodent parasites expressing *P. falciparum* and *P. vivax* pre-erythrocytic antigens have been used to analyse protective immune responses induced by *P. vivax* or *P. falciparum* vaccines in vivo in mice. These chimeric parasites have been used to assess the protective immune responses induced by vaccination that influence sporozoite invasion of hepatocytes both in vitro and in vivo and the removal of infected hepatocytes in vivo [25]. For example, chimeric rodent malaria parasites have been generated where the endogenous *csp* gene has been replaced either with *pfcsp* or different *pvcsp* alleles. These chimeric parasites produce sporozoites that are infectious to rodent hepatocytes in vivo and human hepatocytes in culture [25].

Based on the studies with chimeric rodent parasites we reasoned that the availability of chimeric *P. falciparum* parasites that express *P. vivax* antigens would open up possibilities to analyse protective immune responses induced by vaccination using *P. vivax* antigen-based vaccines in CHMI bypassing the need for *P. vivax* parasite production and measures to ensure that *P. vivax* hypnozoites are removed. As a proof of concept two chimeric *P. falciparum* parasites were generated using CRISPR/Cas9 gene editing methodologies, where the *pfcsp* gene was replaced by one of the two major *pvcsp* alleles, VK210 and VK247 [26]. These chimeric lines, *pf*-*pvcsp*(*vk210)* and *pf*-*pvcsp*(*vk247*), had wild type-like blood stage development and produced normal numbers of oocysts. Unlike the absence of sporozoite formation in *pfcsp* deletion parasites, sporozoite formation did occur inside oocysts of both chimeric lines; however between 50 and 90% of the oocysts degenerated before sporozoite formation and no sporozoites were detected sporozoites in salivary glands. The lack of complete functional complementation was unexpected, since chimeric rodent *Plasmodium berghei* parasites expressing *Pv*CSP-VK210 and *Pv*CSP-VK247 are able to produce salivary gland sporozoites in *An. stephensi* mosquitoes that are infective to mice [27, 28]. The findings in this study and the species-specific features of CSP that may govern full maturation and development of sporozoites of the two human malaria-parasite species are discussed.

## Methods

### *Plasmodium falciparum* and *Plasmodium berghei* parasites and in vitro cultivation of *P. falciparum* blood stages

*Plasmodium falciparum* parasites from the NF54 strain [29] were obtained from the Radboud University Medical Center (Nijmegen, The Netherlands). Parasites were cultured following the standard conditions in RPMI-1640 culture medium supplemented with l-glutamine and 25 mM HEPES (Gibco Life Technologies), 50 mg/l hypoxanthine (Sigma). Culture medium was supplemented with 10% human serum and 0.225% NaHCO_3_. Parasites were cultured at a 5% haematocrit under 4% O_2_, 3% CO_2_ and 93% N_2_ gas-conditions at 75 rpm at 37 °C in a semi-automated culture system in 10 ml flasks (Infers HT Multitron and Watson Marlow 520U). Fresh human serum and human red blood cells (RBC) were obtained from the Dutch National Blood Bank (Sanquin Amsterdam, the Netherlands; permission granted from donors for the use of blood products for malaria research and microbiology test for safety). RBC of different donors were pooled every 2 weeks, washed twice in serum free RPMI-1640 and suspended in complete culture medium to 50% haematocrit. Human serum of different donors were pooled every 4–6 months and stored at − 20 °C until required.

In addition, *P. falciparum* gametocytes cultures were generated using standard culture conditions (see above) with some modifications [30]. Briefly, parasites from asexual stage cultures were diluted to a final parasitaemia of 0.5% and cultures were followed during 14 days without refreshing RBC. After 9 days these cultures were treated with 50 mM of N-acetyl-D-glucosamine (Sigma) to kill asexual stages and to enrich for gametocytes. At day 14 the cultures were analysed for mature, stage V, gametocytes.

Four different mutant lines of the rodent parasite *P. berghei* were used that have been previously reported. (i) A transgenic reference line of *P. berghei* ANKA, expressing the fusion protein GFP-Luciferase (line 676m1cl1; *PbΔp230p*; RMgm-29; https://www.pberghei.eu) [31]; (ii) A mutant that expresses *P. vivax* CSP (VK210 allele). In this mutant the *pbcsp* gene has been replaced with the *pvcsp*-*vk210* gene (line 2196cl1; RMgm-4136; https://www.pberghei.eu); [27]; (iii) A mutant that expresses *Pv*CSP (VK247 allele). In this mutant the *pbcsp* gene has been replaced with the *pvcsp*-*vk247* gene (line 2199cl1; RMgm-4137; https://www.pberghei.eu; [27];

### Generation and selection of the chimeric lines pf-pvcsp(vk210) and pf-pvcsp(vk247)

In order to create *pf*-*pvcsp*(*vk210)* and *pf*-*pvcsp*(*vk247)*, the previously described pLf0019 construct, containing the *cas9* gene was used [32] and 2 different sgRNA-donor DNA containing plasmids, pLf0042 (targeting *Pf*CSP and containing the *pvcsp*-*vk210* gene) and pLf0043 (containing the *pvcsp*-*vk247*). The pLf0019 construct contains a *blasticidin* (BSD) drug-selectable marker cassette and both sgRNA-donor DNA constructs (pLf0042 and pLf0043) contain a h*dhfr* drug-selectable marker cassette for selection with WR99210. To generate pLf0042 and pLf0043, plasmid pLf0033 (see Additional file 1) was modified by introducing two homology regions targeting *Pfcsp* (PF3D7_0304600). Homology region 1 (HR1) was amplified using primers P1/P2 and homology region 2 (HR2) with P3/P4 from *P. falciparum* NF54 genomic DNA (see Additional file 2 for primer details). HR1 was cloned in pLf0033 using restriction sites *kpn*I/*EcoR*I and HR2 using *EcoR*I/*Aat*II. The *pvcsp* alleles *pvcsp*-*vk210* (GenBank accession number P08677; Belem strain) and *pvcsp*-*vk247* (GenBank accession number M69059.1; Papua New Guinea strain) were amplified from existing plasmids PbG01-PvCSP-vk247 and PbG01-PvCSP-vk210 [27] using the primers P5/P6 and cloned into pL0033 containing the HR using restriction sites *EcoR*V/*EcoR*I, resulting in intermediate plasmids pLf0040 and pLf0041 (*pvcsp*-*vk210* and *pvcsp*-*vk247*; see Additional file 1). An additional plasmid, AS301 (see Additional file 1) was used to clone the guide sgRNA (AS301-sgRNA2) specific for *pfcsp*. The sgRNA sequence was identified using the Protospacer software (alphaversion; https://sourceforge.net/projects/protospacerwb/files/Release/) and was amplified using the primers P7/P8. This sgRNA was selected based on the best-off targets hits score throughout the genome given by Protospacer and the total number of mismatches of the sgRNA with respect to the PAM site. A 20 bp guide sgRNA sequence, flanked on both sides by a 15 bp DNA sequence necessary for In Fusion cloning (HD Cloning Kit; Clontech), was annealed and used to replace the BtgZI adaptor as previously described [33]. The construct was then digested with *Bln*I and *Nru*I to evaluate the successful cloning of the sgRNA and later confirmed by Sanger sequencing using primers P9/P10. Finally, the AS301-sgRNA2 was digested with EcoR*V*/Apa*I* and cloned into the vectors pLf0040 and Plf0041 using the restriction sites *Stu*I/*Apa*I resulting in the final constructs pLf0042 and pLf0043 (see Additional file 1).

Plasmids for transfection were isolated from 250 ml cultures of *Escherichia coli* XL10-Gold Ultracompetent Cells (Stratagene) by maxi-pep (using HiSpeed^®^ Plasmid Maxi Kit (Qiagen^®^)) to generate 25–50 µg of DNA used per transfection. Transfections of *P. falciparum* NF54 parasites were performed using ring stage parasites obtained from cultures with a parasitaemia of 6–15% that were synchronized by 5% d-sorbitol treatment 2 days before transfection [34]. Infected RBC were pelleted by centrifugation (1150*g*, 5 min) and 300 µl of the pelleted cells were transferred to a 0.2 cm cuvette and mixed with ~ 50 µg of each circular plasmid (Cas9 and sgRNA/Donor DNA constructs) in 100 µl cytomix [35]. Electroporation was performed with a single pulse (310 V and 950 µF) in the Biorad Gene Pulser Xcell electroporator (including CE- and PC module) and cells were immediately transferred in a 10 ml culture flask and cultures were maintained under standard conditions in the semi-automated culture system (see above). Selection of transformed parasites was performed by applying ‘double’ positive selection 24 h after transfection using the drugs WR99210 (2.6 nM) and BSD (5 µg/ml). For WR99210 100 µl of a stock solution (2.6 µM) was added to 100 ml complete culture medium resulting in a final concentration of 2.6 nM. To prepare the WR99210 stock-solution WR99210 was dissolved in DMSO (100 mM). For BSD 50 µl of a stock solution (10 mg/ml) was added to 100 ml complete culture medium resulting in a 5 µg/ml final concentration. Drug pressure in the cultures was maintained until thin blood-smears were parasite-positive (usually after 14–26 days). Positive selection will select for the parasites that were transfected successfully with both plasmids (Cas9 and sgRNA/Donor constructs). Subsequently, both drugs were removed from the cultures for 2–4 days, followed by applying negative selection by addition of 5-Fluorocytosine (5-FC; 130 µl of a stock solution (0.77 mM) in 100 ml complete medium with a final concentration of 1 µM; [36]) in order to eliminate parasites that retained the crRNA/Donor construct as episomal plasmid and enrich for the transfected parasites containing the donor DNA integrated into the genome. Negative drug pressure in the cultures was maintained until thin blood-smears were parasite-positive (usually after 7 days). After negative selection infected RBC (iRBC) were harvested from cultures with a parasitaemia of 4–10% for genotyping by diagnostic PCR and Southern blot analysis. Subsequently, selected parasites were cloned by limiting dilution.

### Generation and selection of the PfΔcsp line

In order to create the *Pf*Δ*csp* line, a new plasmid (pLf0070: that contain both the crRNA and the Cas9-expression cassette, was modified in order to introduce sgRNAs against *Pfcsp.* This plasmid, kindly obtained from Dr. Marcus Lee (Wellcome Trust Sanger Institute, Wellcome Genome Campus, Hinxton, Cambridgeshire, UK) is based on plasmid pDC2-cam-Cas9-U6.2-hdhfr [37] with a smaller U6 cassete (693 bp) and with the Cas9 gene harmonized to *P. falciparum*. Two plasmids with two different sgRNAs (026 and 012) were generated. Briefly, pLf0070 was digested with *Bbs*I and sgRNA026 was cloned using the primers P11/P12 and sgRNA012 using the primers P13/P14. The primers (100 µM each primer) were phosphorylated with T4 polynucleotide kinase (10 Units per reaction) during 30 min at 37 °C, followed by an annealing program of 5 min incubation at 94 °C and a ramp down to 25 °C at 5 °C per min, and subsequently ligated into the vector using T4 ligase (5 units) resulting in plasmid pLf0071 and plasmid pLf0072 (see Additional file 1). A second DNA donor plasmid was generated by replacing the *Pvcsp*-*vk210* gene of the pLf0040 construct (see above) by an mCherry expression cassette obtained from an intermediate plasmid pLf0055 (see Additional file 1). In this cassette mCherry is under control of the promoter region of *gapdh* (GeneID PF3D7_1462800) and the 3′UTR of histidine-rich protein II(GeneID PF3D7_0831800). The complete mCherry expression cassette was removed from pLf0055 by digestion with the *EcoR*I/*Nru*I and cloned into pLf0040 digested with *EcoR*I/*EcoR*V resulting in the DNA donor vector pLf0083 (see Additional file 1). This donor DNA construct has a drug selectable marker cassette containing a fusion of the positive selectable marker *hdhfr* and the negative selectable marker y*fcu* (*yeast cytosine deaminase/uridyl phosphoribosyl transferase*).

Transfections of *P. falciparum* NF54 parasites was performed by spontaneous plasmid uptake from plasmid-loaded red blood cells cultured under static conditions [38]. Briefly, 300 µl of pelleted, uninfected RBC were transferred to a 0.2 cm cuvette and mixed with 50 µg of both sgRNA constructs (25 µg of pLf0071 and 25 µg of pLf0072) and 50 µg of the donor construct (pLf0083) suspended in 200 µl of cytomix. Electroporation was performed as described in the previous section. After electroporation of the uninfected RBC, iRBC containing *P. falciparum* NF54 parasites were added to a concentration of 0.1%. Selection of transformed parasites was performed when cultures reached a parasitaemia of 3% (after approx. 3 days) with 100 µl of WR992010 (2.6 nM) during a period of 6 days. Subsequently the drug was removed and parasites were harvested at 0.8% of parasitaemia for mCherry fluorescence microscopy analysis to determine the ratio of wild type and mutant parasites present in the population. Parasites were collected from cultures that contained > 80 mCherry-positive parasites (at a 4–10% parasitaemia) for genotyping by diagnostic PCR and Southern blot analysis and for cloning (see next section).

### Cloning of transfected *P. falciparum* parasites

Based on the PCR confirmation of the integration, the transfected parasites were cloned by the method of limiting dilution as previously described [39] with minor modifications. Briefly, infected RBC from cultures with a 4 to 10% parasitaemia were diluted with uninfected RBC to 10^5^ iRBC/100 µl in 2 ml culture medium (1% haematocrit and 20% serum). Serial dilutions were then performed with uninfected RBC in complete medium (1% haematocrit and 20% serum) and cultured in a total volume of 100 µl incubated in 96 well plates, resulting in 8 rows with the following numbers of iRBC per well: 100, 10, 5, 2.5, 1.25, 0.6, 0.3, 0.15. Plates were incubated in a Candle Jar at 37 °C and culture medium was changed every other day. Every 5 days RBC were added resulting in an increase of the haematocrit from 1 to 5%. Between days 10–14 samples were collected for thick smear analysis from the rows with the highest numbers of iRBC/well; 50 µl medium was removed and from the remaining culture 5 µl was used directly for preparing thick smears. At day 21 thick smears were made from all rows. Clones were selected from dilutions/row with less than 30% of the wells parasite positive. These clones were transferred in 10 ml culture flasks at 5% haematocrit under standard culture conditions (see previous sections) in the semi-automated culture system for collection of parasites for further genotype and phenotype analyses.

### Genotyping of the *pf*-*pvcsp*(vk210), *pf*-*pvcsp*(vk247) and *pfΔcsp* lines

For genotyping of the chimeric *pf*-*pvcsp* lines and the *pf*Δ*csp* line diagnostic PCR and Southern blot analysis of digested DNA were performed from material isolated from iRBC obtained from 10 ml cultures (parasitaemia 3–10%), pelleted by centrifugation (1150*g*; 5 min.). RBC were then lysed with 5–10 ml of cold (4 °C) erythrocyte lysis buffer (10 × stock solution 1.5 M NH_4_Cl, 0.1 M KHCO_3_, 0.01 M Na_2_EDTA; pH 7.4; [34]) and parasites were treated with RNAse and proteinase-K before DNA isolation by standard phenol–chloroform methods. Correct integration of the donor constructs was analysed by standard and long-range PCR (LR-PCR). In brief, for the chimeric *pf*-*pvcsp* lines integration of the *pvcsp* cassettes was confirmed by LR-PCR using the primers P15/P16 (and analysed by *EcoR*V digestion). The PCR-amplified product was cloned in a TopoTA vector for sequencing (see Additional file 3 for details of the primers and Additional file 2 for sequence data). The LR-PCR fragments were amplified using KOD Hot start polymerase following standard conditions with an annealing temperature of 53.5 °C for 15 s and an elongation step of 68 °C for 9 min. For the *Pf*Δ*csp* line, 5′-integration PCR was performed using the primers P15/P19 and to confirm the presence of the mCherry gene PCR was performed with the primers P20/P21. The PCR fragments were amplified using Go-taq^®^ DNA polymerase (Promega) following standard conditions with an annealing temperature of 56 °C for 20 s and a elongation step of 72 °C for 4 min. All other PCR settings were according to manufacturer’s instructions.

**Fig. 1:**
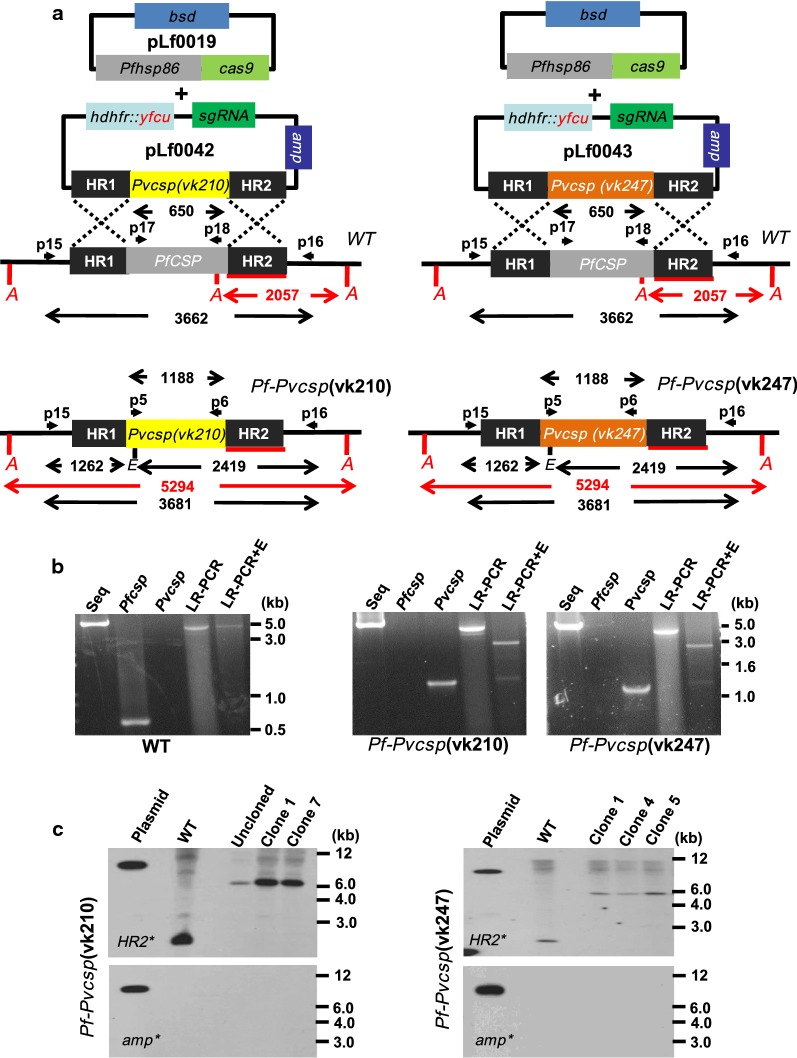
Generation and genotyping of two chimeric *P. falciparum* parasites (*Pf*-*pvcsp*). **a** Two *Pf*-*pvcsp* parasite lines were generated using CRISPR/Cas9 methodology. The coding sequence (CDS) of *Pfcsp* gene was replaced by insertion of the *Pvcsp*(vk210) and *Pvcsp*(vk247) CDS using donor-DNA plasmids pLf0042 and pLf0043. A schematic representation of the *Pfcsp* locus before and after insertion of the construct showing the location of the restriction sites (*A: Ava*II, *E: EcoR*V), sizes (in bp) of restriction fragments (red for Southern blot analysis), location of primers (p), PCR amplicons and sizes (in bp) of the fragments (in black (**b**, **c**). HR1, HR2: *Pfcsp* homology (targeting) regions. The figure is not shown to scale. Primer sequences can be found in Additional file 2. **b** Diagnostic PCR and long-range PCR (LR-PCR) confirming the correct integration of the *Pvcsp* CDS into the *PfCSP* locus. Diagnostic PCR: *Pfcsp* open reading frame (lane 2; primers p17/p18); *Pvcsp* open reading frame (lane 3; primers p5/p6); *P. falciparum* s*equestrin* gene as a control gene (lane 1; primers p22/p23). LR-PCR: products were run undigested or digested with *EcoR*V (LR-PCR + E) in order to confirm double cross-over recombination. LR-PCR (lane 4) of cloned parasites of *Pf*-*pvcsp*(vk210)(cl7; primers p15/p16), *Pf*-*pvcsp*(vk247)(cl5; primers p15/p16) and WT. LR-PCR fragments digested with *EcoR*V (lane 5) for confirmation of double cross-over integration. **c** Southern blot analysis of *Ava*II restricted DNA of WT and chimeric *Pf*-*Pvcsp* parasites confirms the specific integration of the *Pvcsp* genes into the *pfcsp* gene locus. DNA was hybridized with a probe targeting the homology region 2 of *pfcsp* (upper panels; HR2; primers p3/p4; see **a** In addition, to show absence of donor-DNA plasmid and single cross-over events, DNA was hybridized with a probe for the ampicillin gene (lower panels; intermediate donor-DNA plasmid pLf0040 digested with *Aat*II and *Pvu*I). The hybridization pattern observed with the HR2 probe identified the expected different-sized DNA fragments in WT and *pf*-*pvcsp* parasites (2057 and 5294 bp)

Southern blot analysis for the chimeric *pf*-*pvcsp* lines was performed with genomic DNA digested with *Ava*II (4 h at 37 °C) in order to confirm integration of the replacement of *pfcsp* by the *pvcsp* genes Fig. 1c). Digested DNA was hybridized with probes targeting the *Pfcsp* homology region 2 (HR2), amplified from NF54 genomic DNA by PCR using the primers P3/P4 and a second probe targeting ampicillin (Amp) gene, obtained by digestion of the intermediate plasmid pLf0040 with *Aat*II/*Pvu*I (550 bp). For Southern blot analysis of *Pf*Δ*csp*, genomic DNA was digested with *Ava*II and *Xho*I (4 h at 37 °C) and digested DNA was hybridized with the same probes used with the *Pf*-*Pvcsp* lines (HR2 and Amp probes see Additional file 7C).

### Phenotype analysis of *P. falciparum* parasites: blood stages, gametocytes, oocysts and sporozoites

The growth rate of asexual blood stages of the *pf*-*pvcsp* and *pf*Δ*csp* lines was monitored in 10 ml cultures maintained in the semi-automated culture system under standard culture conditions (see above). Briefly, a 0.5% parasitaemia culture was established in complete culture medium at a haematocrit of 5%. Medium was changed twice daily and the culture maintained for a period of 5 days without refreshing RBC. For determination of the course of parasitaemia, triplicate samples of 100 µl were collected daily from all cultures and cells pelleted by centrifugation (9485*g*; 30 s) and stained with Giemsa. mCherry expression of in *Pf*Δ*csp* blood stages was analysed by standard fluorescence microscopy. In brief, 200 µl samples were collected from 10 ml cultures with a parasitaemia between 4 and 10% and stained with the DNA-specific dye Hoechst-33342 by adding 4 µl of a 500 µM stock-solution (final concentration 10 µM) for 20 min at 37 °C. Subsequently, a 5 µl drop was placed on a microscopic slide (mounted under a cover slip) and fluorescence in live iRBC analysed using a Leica fluorescence MDR microscope (100× magnification). Pictures were recorded with a DC500 digital camera microscope using Leica LAS X software and with the following exposure times: mCherry 0.6 s; Hoechst-33342 0.136 s; bright field 0.62 s (1× gain).

Gametocyte production by the *pf*-*pvcsp* and *pf*Δ*csp* lines was analysed in gametocyte cultures, established as described in the previous sections. To activate gametocytes for exflagellation 20 µl samples of the *P. falciparum* stage V gametocyte cultures at day 14 were diluted 1:1 with FCS at room temperature. Gametes and exflagellation centres were examined and quantified 10–20 min after activation using a Bürker cell counter. For analysis of mosquito stages (oocysts and sporozoites) of the chimeric *pf*-*pvcsp* lines, *An. stephensi* were infected using the standard membrane feeding assay (SMFA) [40, 41]. Oocysts were analysed between day 8 and 14 for sporozoite production and the percentage of degenerated oocyst determined. Oocysts were qualified as degenerated based on the following criteria: no sporozoite formation visible and oocyst cytoplasm vacuolated. Salivary gland sporozoites were counted at day 14 and 21 post feeding. For counting sporozoites, salivary glands from 30 to 60 mosquitoes were dissected and homogenized using a grinder in 100 µl of RPMI pH 7.2 and sporozoites were analysed in a Bürker cell counter using phase-contrast microscopy.

### Analysis of PvCSP expression in oocyst-derived sporozoites of the *pf*-*pvcsp*(vk210) and *pf*-*pvcsp*(vk247) lines

To analyse CSP expression in oocyst-derived sporozoites of the *pf*-*pvcsp* lines by immunofluorescence microscopy, midguts from 30 to 60 *An. stephensi* mosquitoes were collected in an eppendorf tube at day 10 after feeding in RPMI-1640 medium containing 3% BSA. Midguts were mechanically crushed using a grinder and centrifuged with low speed (62*g*) for 3 min at 4 °C. Subsequently, the supernatant containing oocyst-derived sporozoites was collected for fluorescence microscopy and samples (20 µl) were placed on a 8-well black cell-line diagnostic microscope slide (Thermo Scientific), dried for 10 min, and fixed with 4% paraformaldehyde for 30 min at room temperature. After fixation the slides were washed three times with 1× PBS and permeabilized with 20 µl of 0.5% triton in 1× PBS and then blocked with 10% of FCS in 1× PBS for 1 h. Fixed cells were washed with 1× PBS and incubated with monoclonal antibodies against PvCSP(vk210) (mouse, anti-PvCSP-VK210 MAb (MR4); 1:200 dilution of 109 µg/ml stock solution [27]) PvCSP-(vk247) (mouse, *anti*-PvCSP-VK247 MAb (MR4); 1:200 dilution of 125 µg/ml [27]), PfCSP (mouse, anti-PfCSP (210 A) MAb(MR4); 1:200 dilution of 8 µg/ml stock solution REF) and PfHSP70 (rabbit, anti-PfHSP70; 1:200 dilution of 100 µg/ml stock solution StressMarqBiosciences) for 1 h at room temperature. Subsequently, cells were rinsed three times with 1× PBS and incubated with the secondary antibodies Alexa FLuor^®^488/594-conjugated chicken anti-mouse and anti-rabbit (Invitrogen Detection technologies at 1:200). Finally, the cells were washed again three times with 1× PBS and stained with the DNA-specific dye Hoechst-33342 at a final concentration of 10 µM. Fixed cells were covered with 1–2 drops of an anti-fading agent (Vectashield), and a coverslip placed on the cells and sealed with nail polish. Stained cells were analysed for fluorescence using a Leica fluorescence MDR microscope (100× magnification). Pictures were recorded with a DC500 digital camera microscope using Leica LAS X software with the following exposure times: Alexa 488: 0.7 s; Alexa 594: 0.6 s Hoechst 0.136 s; bright field 0.62 s (1× gain).

### Phenotype analysis of *Plasmodium berghei* parasites: oocysts, sporozoites and sporozoite infectivity

Feeding of *An*. *stephensi* mosquitoes with *P. berghei* parasites, determination of oocyst production and sporozoite collection were performed as described [27]. Determination of parasite liver load by in vivo imaging and determination of the prepatent period in mice after intravenously injection of 1000 salivary gland sporozoites was performed as described [27].

### Statistics

Data were analysed using GraphPad Prism software package 5.04 (GraphPad Software, Inc). Significance difference analyses between WT, *pf*-*pvcsp* and the *pf*Δ*csp* lines was performed using the unpaired Student’s *t*-test.

## Results

### Generation of two chimeric *P. falciparum* parasites lines expressing PvCSP-210 or PvCSP-247

Using CRISPR/Cas9 gene editing, two chimeric *P. falciparum* parasites lines were created, *pf*-*pvcsp*(vk210) and *pf*-*pvcsp*(vk247), where the *P. falciparum csp* gene has been replaced by either one of the two major *P. vivax csp* alleles, VK210 and VK247. A previously described Cas9 construct (pLf0019), containing the Cas9 expression cassette with a *blasticidin* (BSD) drug-selectable marker cassette [32], was used in combination with two sgRNA donor-DNA containing plasmids, pLf0042 and pLf0043. These constructs are used to target the *pfcsp* gene locus and as ‘DNA-donor’ sequences they replace *pfcsp* with either *pvcsp*-*vk210* or the *pvcsp*-*vk247* full-length gene coding sequences (Fig. 1). The coding sequences of *pvcsp*-*vk210* (GenBank accession number P08677; Belem strain) and *pvcsp*-*vk247* (GenBank accession number M69059.1; Papua New Guinea strain) were amplified from existing plasmids P. bergheiG01-PvCSP-vk247 (pL1943) and P. bergheiG01-PvCSP-vk210 (pL1942) [27]. The two homology regions targeting *pfcsp* (PF3D7_0304600) were amplified from genomic *P. falciparum* DNA (NF54 strain). Both constructs contain a *hdhfr*-*yfcu* drug-selectable marker cassette.

Transfections of *P. falciparum* NF54 parasites were performed using synchronized ring stage parasites that were transfected with ~ 50 µg of each circular plasmid (Cas9 and sgRNA/donor-DNA constructs; see Additional file 1) and selection of transformed parasites containing both plasmids (Cas9 and sgRNA/donor-DNA constructs) was performed by applying ‘double’ positive selection with the drugs WR99210 and BSD, until parasites were detectable by thin blood-smear analysis (between day 14 and 26 post transfection). Subsequently, parasites were cultured for 2–4 days without drugs, followed by applying negative selection to eliminate parasites that retained the transfection constructs (i.e. donor-DNA) as episomal plasmids and to enrich for transfected parasites in which the donor-DNA construct has integrated into the parasite genome. Genotyping of selected parasite populations by long-range PCR revealed that both *pvcsp*(vk210) and *pvcsp*(vk247) cassettes had integrated into the *P. falciparum* genomes (Fig. 1b) and Southern blot analysis of cloned lines confirmed correct integration of the constructs (Fig. 1c). Phenotype analyses, as described below, were performed using *pf*-*pvcsp*(vk210) clone 7 and *pf*-*pvcsp*(vk247) clone 5. Sequence analysis of the long-range PCR products confirmed the correct sequence of *pvcsp* genes and replacement of the *pfcsp* gene in both *pf*-*pvcsp* lines (see Additional file 3).

### *Pf*-*Pv*CSP210 and *Pf*-*Pv*CSP210 parasites form oocysts but salivary gland sporozoites are absent in *An. stephensi* mosquitoes

The growth of asexual blood stages in cultures and gametocyte production of both *pf*-*pvcsp* lines was comparable to that of the parental *P. falciparum* NF54 wild type (WT) parasite strain (see Additional file 4, Table 1). Gametocyte cultures of the *pf*-*pvCSP* lines produced WT-like numbers of mature, stage V, gametocytes of both sexes. Mature male gametocytes of both lines underwent exflagellation upon activation and were able to form male gametes (Table 1).

**Table 1:** Gametocyte, oocyst and sporozoite production in WT, *pf*-*pvcsp(vk210)*, *pf*-*pvcsp(vk247)* and *Pf*Δ*csp*

Lines	No of gametocytes stage V male/female mean (SD)^a^	No. of exflagellation mean (SD)^b^	No. of oocyst mean (range)^c^	No of sporozoites^d^
***WT***				
*NF54* (n = 4)	m: 0.6 (0.2) f: 1.3 (0.3) (3 exp.)	1436 (191) (3 exp.)	28.2 (13–54) (4 exp)	9–20 K (4 exp)
***pf*** **-** ***pvcs*** **(vk210)**				
*0050cl7* (*n *= *5*)	m: 0.6; f: 1.1 (1 exp.)	2030 (916) (3 exp.)	44.2 (30–63) (5 exp)	Negative
*0050cl2* (*n *= *1*)	ND	ND	9.1 (1 exp.)	Negative
***pf*** **-** ***pvcs*** **(vk247)**				
*0041cl5* (*n *= *3*)	m: 0.8; f:1.5 (1 exp.)	460 (34) (3 exp.)	23.1 (17–40) (3 exp.)	Negative
***Pf*** **Δ** ***csp***				
*0113cl1* (*n *= *1*)	ND	875 (1 exp.)	4.6	Negative
*0113cl3* (*n *= *2*)	m: 0.6 f: 1.1 (1 exp.)	1010 (388) (3 exp.)	2.2 and 3.2 (2 exp.)	Negative

^a^Mean percentage of stage V male (m) and female (f) gametocytes (per 100 red blood cells) in day 14 cultures in 2–7 experiments (exp.)

^b^Mean number of exflagellating male gametocytes (per 10^5^ red blood cells) at 10–20 min after activation of day 14 gametocyte cultures (*sd* standard deviation)

^c^Mean number of oocyst per mosquito at day 9–10 after feeding. Range corresponds to the mean number of retorts in multiple experiments (1–5 exp. per line; 10–30 mosquitoes per exp.)

^d^Mean number of salivary gland sporozoites per mosquito at day 21 after feeding. Range corresponds to the mean number of sporozoites in multiple experiments (1–5 exp. per line; 20–30 mosquitoes per exp.)

*Anopheles stephensi* mosquitoes were fed with either WT gametocytes or gametocytes of the *pf*-*pvcsp* lines using the standard membrane feeding assay and the number of oocysts in mosquito midguts was determined at day 10 post infection and the presence of sporozoites in the haemocoel and salivary glands was analysed at day 14 and day 21. Dissection of WT and *pf*-*pvcsp* infected mosquitoes revealed that all lines produced comparable numbers of oocysts; however, no sporozoites were detected in salivary glands in mosquitoes infected with either of the *pf*-*pvcsp* lines (Table 1). Moreover, no sporozoites from the *pf*-*pvcsp* lines were found in haemocoel fluid after mosquito dissection; in contrast, when WT infected mosquitoes were dissected many sporozoites were observed in haemocoel fluid.

These observations indicate that the chimeric parasites expressing PvCSP are unable to produce sporozoites that are competent in invading salivary glands.

### *pf*-*pvcsp* lines produce sporozoites in oocysts but most oocysts degenerate before sporozoite formation and release of sporozoites

**Fig. 2:**
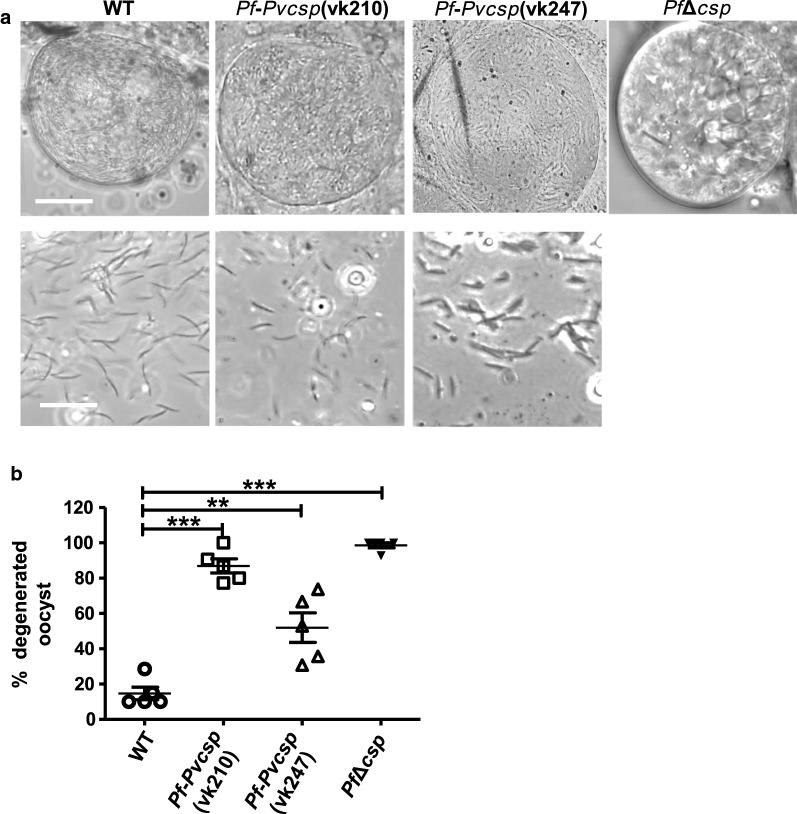
Oocyst and sporozoite formation of two chimeric *P. falciparum* parasite lines (*pf*-*pvcsp*) and a PfCSP knockout line (*Pf*Δ*csp*). **a** Light microscope pictures of oocysts at day 10 after feeding gametocytes to *Anopheles stephensi* mosquitoes. Upper panel *pf*-*pvcsp* and wild type *P. falciparum* (WT) oocyst in which sporozoite formation occurs (see Additional file 5 for pictures of *pf*-*pvcsp* oocyst that degenerate before sporozoite formation). No sporozoite formation was observed in *Pf*Δ*csp* oocysts. Scale bar, 20 µm. Lower panel: free *pf*-*pvcsp* sporozoites that are released from oocysts by application of force to oocysts in dissected midguts. Scale bar, 20 µm. **b** Percentage of degenerated oocyst in *An. stephensi* mosquitoes (n = 5) at day 10 after feeding (**P = 0.0035, ***P = <0.0001; unpaired T-test)

For two *Plasmodium* species it has been shown that *csp* deletion mutants can form oocysts; however by light-microscopy, these oocysts are highly vacuolated and do not show signs of sporozoite formation [42, 43]. Therefore, oocyst development of *pf*-*pvcsp* was analysed in greater detail by light microscopy and *Pv*CSP expression was examined by immunofluorescence microscopy. After feeding with both *pf*-*pvcsp* lines, oocysts were readily detected at day 10, in which sporozoite formation occurred (Fig. 2a). However, analysis of oocysts between day 10 and 14 showed that most oocysts started to degenerate before full maturation as shown by the absence of clear sporozoite formation and the presence of large vacuoles in the cytoplasm of maturing oocysts (see Additional file 5). In WT-infected mosquitoes, 30% of the oocysts were characterized as degenerate at day 10, whereas in *Pf*-*Pv*CSP(vk210) and *Pf*-*Pv*CSP(vk247) infected mosquitoes 52 and 87% degenerate oocysts were countedy (Fig. 2b). No spontaneous release of sporozoites from mature *pf*-*pvcsp* oocysts was detected and no sporozoites could be found free in the haemocoel or in salivary glands, even up to day 21 after feeding. Only through the application of force to oocysts in dissected midguts could mechanically liberated oocyst-derived *pf*-*pvcsp* sporozoites be recovered (Fig. 2a).

### Chimeric sporozoites of both *pf*-*pvcsp*(vk210) and *pf*-*pvcsp*(vk247) express PvCSP

**Fig. 3:**
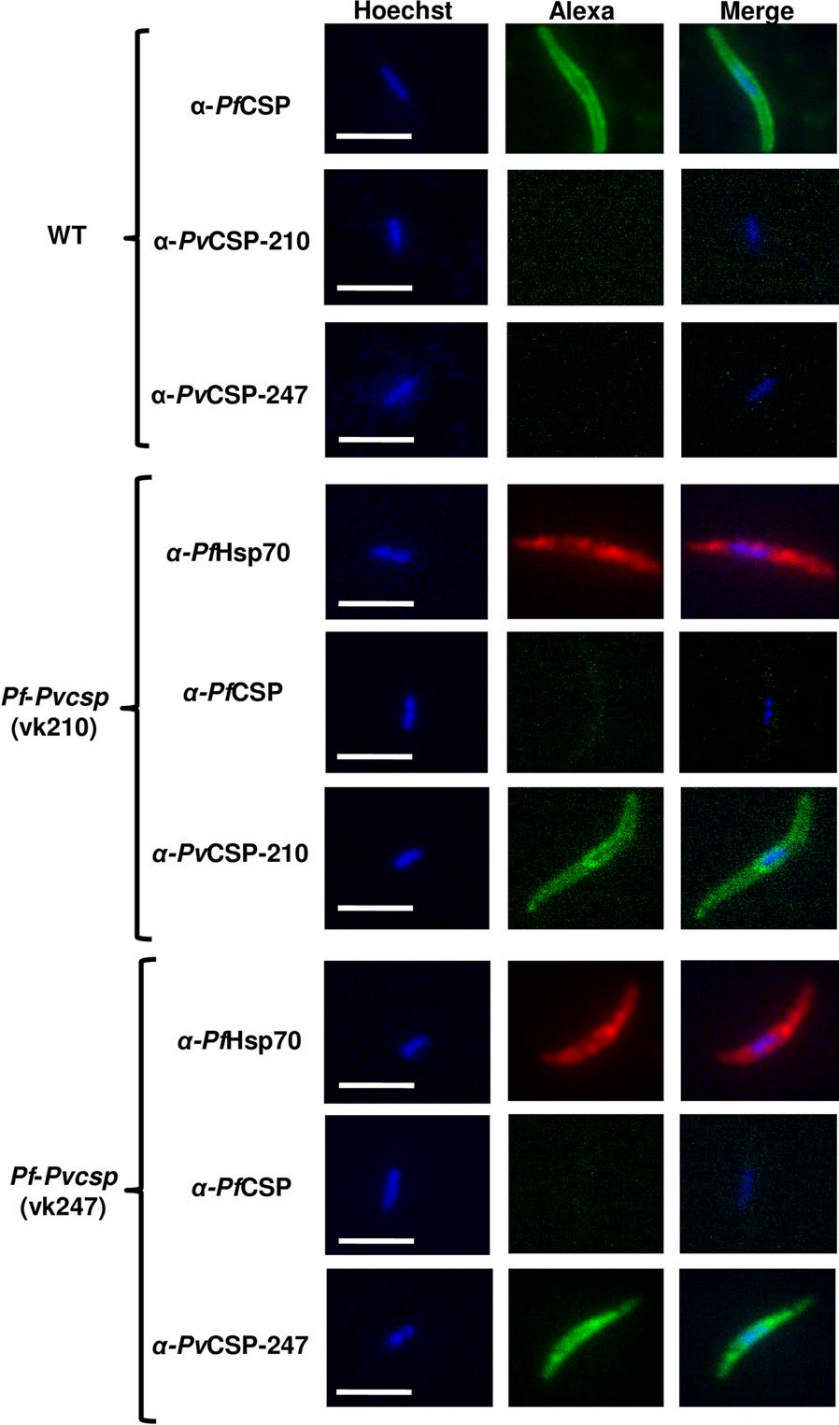
PvCSP(VK210) and PvCSP(VK247) expression in oocyst-derived sporozoites of two chimeric *P. falciparum* parasite lines (*pf*-*pvcsp*). Immunofluorescence analyses of wild type *P. falciparum* (WT) sporozoites and oocyst-derived *pf*-*pvcsp* sporozoites. Fixed sporozoites were labelled with mouse anti-PvCSP-VK210 mAb, anti-PvCSP-VK247mAb and mouse anti-PfCSP antibodies. As a control an antibody against PfHSP70 was used. Secondary conjugated antibodies used: anti-IgG Alexa Fluor^®^ 488 (green) or anti-IgG Alexa Fluor ^®^ 594 (red). Nuclei stained with the DNA-specific dye Hoechst-33342. All pictures were recorded with standardized exposure/gain times; Alexa Fluor^®^ 488 (green) 0.7 s; anti-IgG Alexa Fluor ^®^ 594 (red) 0.6 s; Hoechst (blue) 0.136 s; bright field 0.62 s (1× gain). Scale bar, 7 µm

To analyse expression of PvCSP in *pf*-*pvcsp* sporozoites, oocyst-derived sporozoites were collected by differential centrifugation of extracted, mechanically crushed, infected midguts. These sporozoites were stained with antibodies specific for *Pv*CSP-VK210, *Pv*CSP-VK247 and PfCSP. Sporozoites of *pf*-*pvcsp(vk247)* and *pf*-*pvcsp(vk210)* only reacted with their cognate anti-PvCSP-VK247 or anti-PvCSP-VK210 antibodies and WT sporozoites only with anti-PfCSP antibodies (Fig. 3; Additional file 6). These results indicate that the corresponding PvCSP is expressed in developing oocysts and oocyst-derived sporozoites of the *pf*-*pvcsp* lines and that the failure of formation of fully competent sporozoites is not due to the absence of PvCSP expression.

### A *P. falciparum* NF54 mutant that lacks expression of PfCSP forms oocysts but sporozoite formation is absent

To investigate whether the formation of sporozoites in chimeric *pf*-*pvcsp* oocysts was due to partial complementation of PfCSP by PvCSP, a *P. falciparum* mutant lacking CSP expression was generated. This mutant (*Pf*Δ*csp*) was generated by disrupting the *pfcsp* gene by CRISPR/Cas9 gene editing using a construct (pL0083) that contained an mCherry reporter cassette (under control of the constitutive *gapdh* promoter; see Additional file 7A) flanked by *pfcsp* targeting sequences. This construct is designed to replace the *pfcsp* gene with the mCherry-expression cassette and contains a *hdhfr*-*yfcu* drug-selectable marker cassette. Transfection of *P. falciparum* NF54 parasites was performed by spontaneous plasmid uptake from plasmid-loaded red blood cells cultured under static conditions. Uninfected RBC were mixed with 50 µg of two sgRNA/Cas9 constructs (pLf0071 and pLf0072) and 50 µg of the donor-DNA construct (pLf0083). After electroporation, these uninfected cells were mixed with iRBC containing *P. falciparum* NF54 parasites and selection of transformed parasites was performed with the drug WR99210 during a period of 6 days. Subsequently the drug was removed and parasites were harvested at 0.6–0.8% parasitaemia for mCherry fluorescence microscopy analysis to determine the ratio of WT and mutant parasites expressing mCherry. Parasites were collected from cultures that contained > 80% mCherry-positive parasites (at a 4–10% parasitaemia). Genotyping of selected and cloned mCherry-positive parasites by diagnostic PCR and Southern blot analysis showed integration of the mCherry-expression cassette into the *pfcsp* gene (see Additional file 7B, C).

The growth of asexual blood stages in cultures and gametocyte production of the *Pf*Δ*csp* line was comparable to that of WT *P. falciparum* NF54 parasites (see Additional file 4; Table 1). Gametocyte cultures of the *Pf*Δ*csp* lines produced WT-like numbers of mature, stage V, gametocytes of both sexes. Mature male gametocytes of both lines underwent exflagellation upon activation and were able to form male gametes (Table 1). *Anopheles stephensi* mosquitoes were fed with WT gametocytes and *Pf*Δ*csp* gametocytes using standard membrane feeding. The number of oocysts was determined at day 10 post feeding and the presence of sporozoites in salivary glands analysed at day 14 and 21 after feeding. *Pf*Δ*csp* parasites were able to produce oocysts in the mosquitoes but no sporozoites were detected in salivary glands after feeding (Table 1). Moreover, sporozoite formation was not detected in oocysts (3 exp.; 30 mosquitoes per experiment). All oocysts degenerated before full maturation as shown both by the absence of sporozoite formation and the presence of large vacuoles in the cytoplasm of the oocysts (see Fig. 2; Additional file 5). This oocyst phenotype is comparable to the phenotype observed in *P. berghei* and *Plasmodium knowlesi* mutants lacking CSP expression [42, 43]. The absence of sporozoite formation in *Pf*Δ*csp* oocysts is in support of partial complementation of PfCSP by PvCSP in the *pf*-*pvcsp* lines where sporozoites are formed in a fraction of the oocysts.

### Heterologous CSP replacement and expression in different Plasmodium species

Several rodent malaria parasite mutants have been generated in which their endogenous *csp* gene has been replaced by a *csp* gene from another *Plasmodium* species. In most of these mutants the heterologous CSP protein can complement the function of endogenous CSP (Table 2). In both *Plasmodium yoelii* and *P. berghei*, *P. falciparum* CSP can replace and functionally complement rodent parasite CSP. In addition, multiple chimeric *P. berghei* lines have been generated that express *P. vivax* CSP alleles (Table 2) and these lines produce salivary gland sporozoites that are capable of infecting mice. In *P. berghei* only replacement of PbCSP with CSP of the avian malaria parasite *Plasmodium gallinaceum* did not lead to full complementation and *pb*-*pgcsp* parasites produced strongly reduced numbers of salivary gland sporozoites, which are not infective to mice [44]. Recently, the generation of two chimeric *pb*-*pvcsp* lines, expressing the *pvcsp* (*vk210/vk247*) genes was reported [27]. The *pvcsp*-*210* and *pvcsp247* genes used to generate the chimeric *pf*-*pvcsp* lines described in this study were amplified from the constructs used to generate the rodent *pb*-*pvcsp* lines. The *pb*-*pvcsp* lines produced salivary gland sporozoites in *An. stephensi* mosquitoes that were fully infectious to mice and these lines have been used to analyse protective immunity induced in mice by vaccines that target PvCSP [27]. Oocyst formation and sporozoite production of *pb*-*pvcsp(vk210)* was comparable to that of WT parasites but sporozoite production of *pb*-*pvcsp(vk247)* was reduced (Table 2). Oocyst formation and sporozoite production of these lines was analysed in more detail and confirmed the WT-like formation of oocysts and sporozoites in *pb*-*pvcsp(vk210)* (see Additional file 8). The number of salivary gland sporozoites of *pb*-*pvcsp(vk247)* was however significantly reduced (P < 0.0001***) compared to WT or *pb*-*pvcsp(vk210)* parasites (see Additional file 8). By analysis of oocysts of both lines at day 14 post feeding, we observed increased numbers of degenerated oocysts in the *pb*-*pvcsp(vk247),* a phenotype that was similar to oocysts of the *pf*-*pvcsp* lines, i.e. absence of sporozoite formation and vacuolated oocyst morphology (see Additional file 8). These observations indicate that the differences in numbers of salivary gland sporozoites between the *P. berghei* lines results from a better ability of *P. vivax* CSP(VK210) to complement PbCSP function than PvCSP(VK247).

**Table 2:** Parasite lines of different *Plasmodium* species expressing heterologous or mutated CSP and mutants lacking CSP expression

Plasmodium species	CSP^a^	Oocyst no.^b^	Salivary gland sporozoite no.^b^	Reference; RMgmDB ID^c^	Remarks (mutation, sporozoite phenotype)
Chimeric CSP parasite lines					
*P. berghei*	*Py* CSP	WT	WT	[44]; 75	
*P. berghei*	*Pf* CSP	WT	Reduced (90%)	[52]; 69	
*P. berghei*	*Pf* CSP	WT	Reduced (90%)	342	
*P. berghei*	*Pf* CSP	WT	WT	[53]; 4110	
*P. berghei*	*Pf* CSP	WT	WT	[54]; 4135	
*P. yoelii*	*Pf* CSP	WT	WT	[55]; 1442	
*P. berghei*	*Pv* CSP-210	WT	WT	[27]; 4136	
*P. berghei*	*Pv* CSP-247	WT	WT	[28]; 1443	
*P. berghei*	*Pv* CSP-247	WT	Reduced (30%)	[27]; 4137	
*P. berghei*	*Pg* CSP	WT	Absent	[44]; 74	WT oocyst sporozoite formation
*P. falciparum*	*Pv* CSP-210	WT	Absent	This study	Sporozoite formation in fraction of oocysts
*P. falciparum*	*Pv* CSP-247	WT	Absent	This study	Sporozoite formation in fraction of oocysts
Knock-out CSP parasite lines					
*P. berghei*	–	WT	Absent	[42]; 9	No sporozoite formation
*P. knowlesi*	–	WT	Absent	[43]	No sporozoite formation
*P. falciparum*	–	WT	Absent	This study	No sporozoite formation
Mutated CSP parasite lines (with a sporozoite production phenotype)					
*P. berghei*	Mut. Pb CSP	WT	Absent	[45]; 72	PbCSP with truncated 3′UTR; reduced sporozoite formation in oocysts
*P. berghei*	Mut. Pb CSP	WT	Absent	[50]; 73	Mutations of the C-terminal GPI-anchor. No sporozoite formation in oocysts
*P. berghei*	Mut. Pb CSP	WT	Absent	[46]; 1148	PbCSP lacking repeat region; reduced sporozoite formation in oocysts; no midgut/salivary gland sporozoites
*P. berghei*	Mut. Pb CSP	WT	Absent	[46]; 1149	PbCSP lacking repeat region and NH2 terminus; no sporozoite formation in oocysts
*P. berghei*	*Pb*/*Pg* CSP	WT	Absent	[56]; 770	*pbcsp* replaced by *pgcsp* with repeat region of *pbcsp*

^a^Expression of heterologous CSP or mutated CSP

^b^Oocyst numbers and salivary gland sporozoite numbers in infected *An. stehensi* mosquitoes compared to wild type (WT) infected mosquitoes. *WT* numbers in the same range as WT-infected mosquitoes

^c^Mutant ID in the RMgmDB database: https://www.pberghei.eu

## Discussion

Chimeric *P. falciparum* parasites where the *csp* gene has been replaced with coding sequences of *P. vivax csp*, either *Pvcsp*(vk210) or *Pvcsp*(vk247), do not form salivary gland sporozoites. These observations indicate that *Pv*CSP cannot functionally complement *Pf*CSP. Although *Pv*CSP-expressing sporozoites are formed within oocysts of both chimeric lines, most oocysts degenerate before sporozoite formation and no sporozoites are released from oocysts resulting in the lack of sporozoites in the haemocoel or in salivary glands. The inability of *P. vivax* CSP to functionally complement *P. falciparum* CSP is unexpected as studies in the rodent parasite *P. berghei* have shown that the *P. berghei* CSP can be functionally replaced by CSP from different *Plasmodium* species, including the human *Plasmodium* species, *P. vivax* and *P. falciparum* (Table 2). Chimeric *pb*-*pvcsp* sporozoites expressing the same two PvCSP alleles VK210 and VK247, which were used in this study, are able to invade *An. stephensi* salivary glands and are infectious to mice.

CSP is a multifunctional protein that has an essential role in the formation of sporozoites inside oocysts as well as in sporozoite release, motility and host-cell invasion [9–12]. Mutants of *P. berghei* and the primate parasite *P. knowlesi* lacking CSP expression do form oocysts but sporozoite formation inside oocysts is absent [42, 43]. Maturing oocysts of these *csp*-deletion mutants are highly vacuolated and have no signs of sporozoite formation that could be detected by light microscopy. In addition, highly vacuolated oocysts and absence of sporozoite formation were observed in mosquitoes fed with the *P. falciparum* mutant lacking CSP, *Pf*Δ*csp* generated in this study. These observations confirm the essential role of CSP early in the formation of *Plasmodium* sporozoites and in oocyst maturation.

In contrast to the *Pf*Δ*csp* parasites, where no sporozoite formation was detected in maturing oocysts, we observed *Pf*-*Pvcsp* oocysts with sporozoite formation and we were able to obtain oocyst-derived sporozoites of both chimeric lines. These sporozoites expressed *Pv*CSP as shown by immunofluorescence analysis with antibodies specific for either *Pv*CSP VK210 or VK247. These observations indicate that the *Pv*CSP proteins can be used to initiate sporozoite formation in *P. falciparum* oocysts but are unable to fully complement the function of *Pf*CSP in oocyst maturation and sporozoite development. Despite the formation of typical elongated sporozoites in some oocysts of *Pf*-*Pvcsp* fed mosquitoes, most oocysts exhibit a vacuolated morphology and degenerate before sporozoite formation. Oocyst degeneration was clearly visible from day 10 onwards and between day 10 and 21 no increase in oocysts with sporozoite formation was observed, indicating that the absence of sporozoite formation at day 10 is not the result of a delayed maturation of the oocysts. In addition, no evidence was found for spontaneous release of sporozoites of the oocysts that contained sporozoites and we did not observe haemocoel or salivary gland sporozoites in *Pf*-*Pvcsp* infected mosquitoes up to day 21 post feeding. Free sporozoites were only observed when oocysts were ruptured by applying mechanical forces on these oocysts.

It seems unlikely that the failure of PvCSP to functionally complement PfCSP is due to incorrect expression of the PvCSP proteins in the *pf*-*pvcsp* lines. The same *pvcsp* genes as used for successful complementation of CSP in *P. berghei* [27] were used to replace *P. falciparum csp* and the *pvcsp* genes were amplified from the same plasmids that were used for generation of the *pb*-*pvcsp* lines. In addition, the *pvcsp* gene coding sequence in the genome of the *pf*-*pvcsp* lines was placed under control of the endogenous *pfcsp* promoter and transcriptional terminator sequences to ensure correct timing and level of CSP expression. It has been shown that the 3′ untranslated region (3′-UTR) of *P. berghei csp* plays an important role in accurate CSP expression as truncation of *pbcsp* 3′-UTR results in reduced CSP expression, reduced oocyst sporozoite formation and degeneration of oocysts [45].

The inability of PvCSP to replace the PfCSP function in the chimeric *pf*-*pvcsp* lines is therefore most likely due to sequence differences between these *csp* genes that lead to structural differences of the CSP proteins, which may affect interactions with other parasite proteins that are essential for proper sporozoite formation. These can be protein interactions that influence correct transport of CSP from within the oocyst-cytoplasm to the membrane of developing sporozoites or interactions that affect its localization or maintenance on sporozoites [9–12, 46]. Mutational analyses of *P. berghei* CSP have shown that different regions/sequence motifs of CSP are involved in correct formation of sporozoites in oocysts (Table 2). The overall structure of CSP of different *Plasmodium* species is conserved. CSP is a GPI anchored protein that has a central amino acid repeat region, the sequence and number of repeats varies across *Plasmodium* species. These repeats are flanked by two conserved domains; region I at the N terminus of the repeats, and the thrombospondin repeat (TSR) domain C-terminal to the repeats [9]. The repeat regions of PfCSPs consist of predominantly NANP repeats, which differs in length between individual *P. falciparum* strains [47, 48]. The repeat region of CSP of two major strains of *P. vivax*,VK210 and VK247 are different from PfCSPs to these consists of 10–11 copies of GDRA(A/D)GQPA or ANGA (G/D)(N/D)QPG in CSP-VK210 and CSP-VK247, respectively [49]. The GPI-anchor and the repeat region have been shown to play an essential role in correct sporozoite formation in *P. berghei* oocysts (Table 2). Mutant parasites expressing CSP without GPI-anchor, or with a mutated GPI-anchor, fail to produce sporozoites and the phenotype is similar to mutants that lack CSP expression, i.e. complete absence of sporozoite formation [50]. The repeat region of CSP appears to play a critical role in the formation of *P. berghei* sporozoites. *P. berghei* parasites expressing mutated CSP lacking only the repeat region are affected in the later stages of sporozoite formation [46], a phenotype that more closely resembles the phenotype of the *pf*-*pvcsp* lines. It has been proposed that the CSP repeats play a structural role and their absence may result in misfolding of CSP and this could affect the interaction of CSP with other sporozoite proteins, which are required for final oocyst and sporozoite maturation [46]. However, *P. berghei* parasites that lack the N-terminal portion of CSP, but retain the signal sequence, the repeat region and the C′-terminal region of the protein, can still produce salivary gland sporozoites [51].

The phenotype of chimeric *P. falciparum* expressing PvCSP might therefore be explained by a disturbed interaction of PvCSP with other *P. falciparum* proteins, interactions that are mediated by the repeat sequences and which are necessary for complete maturation and release of oocyst sporozoites. In contrast, it would appear that both PvCSP and PfCSP are able to interact with these CSP interacting proteins in *P. berghei*. *Plasmodium berghei* parasites where PbCSP has been replaced with either PvCSP(VK210) or PvCSP(VK247) produced infectious salivary gland sporozoites, although parasites expressing PvCSP(VK247) produced significantly less salivary gland sporozoites than either WT or PvCSP(VK210)-expressing parasites. Mosquitoes infected with *pb*-*pvcsp(vk247)* contained degenerate oocysts with a vacuolated morphology that resembled *pf*-*pvcsp* parasites. These observations indicate that *Pv*CSP(VK247) is less effective in complementing *P. berghei* CSP function in sporozoite maturation and release from oocysts. However, the *pb*-*pvcsp(vk247)* sporozoites that had invaded the salivary glands were not affected in their ability to infect mice.

Further studies using parasites expressing chimeric CSP molecules, comprised of different domains of PfCSP and PvCSP, are required to reveal which CSP domains are essential for sporozoite maturation and release and can explain the failure of PfCSP complementation by PvCSP. The inability of *pf*-*pvcsp* lines to produce salivary gland sporozoites means that these lines cannot be used for CHMI studies.

## Conclusions

Chimeric *P. falciparum* parasites expressing *P. vivax* circumsporozoite protein fail to produce salivary gland sporozoites. While PvCSP-expressing sporozoites are formed within oocysts, most oocysts degenerate before sporozoite formation, no sporozoites are released from oocysts to results in the absence of sporozoites in either the mosquito haemocoel or salivary glands. Combined, these observations show that while PvCSP can partially complement the function of PfCSP, species-specific features of CSP govern full sporozoite maturation and development in the two human malaria parasites.

The inability of *P. vivax* CSP to functionally complement *P. falciparum* CSP is unexpected as studies in the rodent parasite *P. berghei* have shown that the *P. berghei* CSP can be functionally replaced by CSP from different *Plasmodium* species, including the human *Plasmodium* species, *P. vivax* and *P. falciparum.* It seems unlikely that the failure of PvCSP to functionally complement PfCSP is due to incorrect expression of the PvCSP proteins, as the same *pvcsp* genes were used that were able to successfully complement the function of CSP in *P. berghei*. Further studies on the role of different CSP elements from different *Plasmodium* species and their potential interactions with other *Plasmodium* proteins may not only reveal the species-specific mechanisms that govern sporozoite formation and function but may also provide essential information that can be used to create human infectious chimeric *pf*-*pvcsp* sporozoites.

## Additional files

**Additional file 1.** Vector maps of the *P. vivax csp* genes introduced into *P. falciparum.* **A.** Vector maps of the different plasmids used to generate two chimeric *P. falciparum* parasite lines (*pf-pvcsp*) expressing *P. vivax* CSP(VK210) of CSP(VK247) and a *P. falciparum* line lacking expression of CSP (*Pf*Δ*csp*). See “Methods” section for description and details of the generation of these plasmids.

**Additional file 2.** List of primers used in this study.

**Additional file 3.** Sequence analysis of the *P. vivax csp* genes introduced into *P. falciparum*. Long-range PCR fragments (see Fig. 1) were cloned into pET TOPO TA (Invitrogen) and sequenced. See Additional file 2 for primer sequences used for sequencing the complete fragment. **A.** LR-PCR fragment of parasites of *pf*-*pvcsp*(vk210). **B.** LR-PCR fragment of parasites of *pf*-*pvcsp*(vk247).

**Additional file 4.** Growth of asexual blood stages and mCherry expression in blood stages. Growth of asexual blood stages of two chimeric *P. falciparum* parasite lines (*pf*-*pvcsp*(vk210) and *pf-pvcsp*(vk247), a *P. falciparum* line lacking expression of CSP (*Pf*Δ*csp*) and *P. falciparum* wild type (WT) parasites. Parasites of the different cloned lines were cultured in semi-automated culture system for a period of 7 days. Cultures were initiated with a parasitaemia of 0.5%. Arrows indicate the dilution of the cultures with fresh red blood cells to have a final parasitaemia of 1%. **B.** mCherry-expressing blood stages of *Pf*Δ*csp* parasites where the *csp* gene has been disrupted by insertion of an mCherry expression cassette (see Additional file 5 for details of the generation of *Pf*Δ*csp*). Scale bar, 7 µm.

**Additional file 5.** Degenerated oocysts of two chimeric *P. falciparum* parasite lines (*pf-pvcsp*) and a PfCSP knockout line (*Pf*Δ*csp*). Light microscope pictures of degenerated oocysts at day 10 after feeding gametocytes to *Anopheles stephensi* mosquitoes. These oocysts are classified as degenerate based on the absence of sporozoite formation and vacuolated cytoplasm. See Fig. 2 for *pf-pvcsp* and wild type *P. falciparum* (WT) oocyst in which sporozoite formation occurred. No sporozoite formation was observed in *Pf*Δ*csp* oocysts. Scale bar, 20 µm.

**Additional file 6.** PvCSP(VK210) and PvCSP(VK247) expression in oocyst-derived sporozoites of two chimeric *P. falciparum* parasite lines (*pf-pvcsp*). Immunofluorescence analyses of wild type *P. falciparum* (WT) sporozoites and oocyst-derived *pf-pvcsp* sporozoites. Fixed sporozoites were labelled with mouse anti-PvCSP-VK210 mAb, anti-PvCSP-VK247mAb and mouse anti-PfCSP antibodies. Secondary conjugated antibodies used: anti-IgG Alexa Fluor® 488 (green. Nuclei stained with the DNA-specific dye Hoechst-33342. All pictures were recorded with standardized exposure/gain times; Alexa Fluor® 488 (green) 0.7 s; Hoechst (blue) 0.136 s; bright field 0.62 s (1× gain). Scale bar, 7 µm.

**Additional file 7.** Generation and genotyping a *P. falciparum* mutant line lacking expression of CSP (*Pf*Δ*csp*). **A.** The *Pf*Δ*csp* line was generated using CRISPR/Cas9 methodology. The *pfcsp* gene was replaced by insertion of a mCherry expression cassette (mCherry under control of the of the constitutive *gapdh* promoter) using donor-DNA plasmids pLf0086. A schematic representation of the *pfcsp* locus before and after insertion of the construct showing the location of the restriction sites (*A: Ava*II*)*, sizes (in bp) of restriction fragments (red for Southern blot analysis), location of primers (p), PCR amplicons and sizes of the fragments (in black) used to analyse correct disruption of the *pfcsp* and insertion of the mCherry cassette (**B**, **C**). HR1, HR2: *pfcsp* homology (targeting) regions. The figure is not shown to scale. Primer sequences can be found in Additional file 8. **B.** Diagnostic PCR confirming correct integration of the mCherry cassette into the PfCSP locus. 5′ integration PCR (lane 2; primers p15/p19); *pfcsp* open reading frame (lane 3; primers p17/p18); *P. falciparum sequestrin* as a control gene (lane 1; primers p22/p23); mCherry gene (lane 4; primers P20/P21) of cloned parasites of *Pf*Δ*csp* (cl3) and WT. **C.** Southern blot analysis of *Ava*II/XhoI restricted DNA of WT and *Pf*Δ*csp* parasites confirms the specific integration of the mCherry cassette into the *pfcsp* gene locus. DNA was hybridized with a probe targeting the homology region 2 (upper panels; HR2; primers p3/p4; see (**A**) of *pfcsp*. The hybridization pattern observed with the HR2 probe identified the expected different-sized DNA fragments in WT and *pf-pvcsp* parasites (2057 bp and 4105 bp). In addition to show absence of donor-DNA plasmid and presence of single cross-over events DNA was hybridized with a probe for the ampicillin gene (lower panels; intermediate donor-DNA plasmid pLf0040 digested with *Aat*II and *Pvu*I).

**Additional file 8.** Chimeric rodent parasite lines *pb-pvcsp(*vk210) and *pb-pvcsp(*vk247) are able to produce salivary gland sporozoites that are infectious to mice. A. Upper panel, left: Light microscope pictures of wild type *P. berghei* (WT) and *pb-pvcsp* oocyst at days 7, 10 and 14 after feeding of *An. stephensi* mosquitoes. Black arrows indicate oocyst with sporozoite formation and white arrows indicate degenerated, vacuolated oocyst without sporozoite formation (scale bar, 10 µm). Upper panel, right, Percentage of degenerated oocyst in *An. stephensi* mosquitoes (n = 5) at day 14 after feeding (***P = < 0.0001; unpaired T-test). Lower panel: Oocyst and sporozoite production of wild type *P. berghei* (WT) and *pb-pvcsp* parasites. B. *In vivo* imaging of parasite liver loads at 44 h after injection of mice with salivary gland sporozoites of wild type *P. berghei* (WT) and *pb-pvcsp* parasites. Left panel: luminescence signals in the different groups of mice. Right panel: quantification of the bioluminescence signals in the different groups of mice measured as Relative Luminescence Units (RLU).
