# Supplementary figures and images for: Chimeric Plasmodium falciparum parasites expressing Plasmodium vivax circumsporozoite protein fail to produce salivary gland sporozoites
Source: Malar J. 2018 Aug 9;17:288. doi: 10.1186/s12936-018-2431-1 (PMC6085629; doi:10.1186/s12936-018-2431-1)

Intermediate plasmids

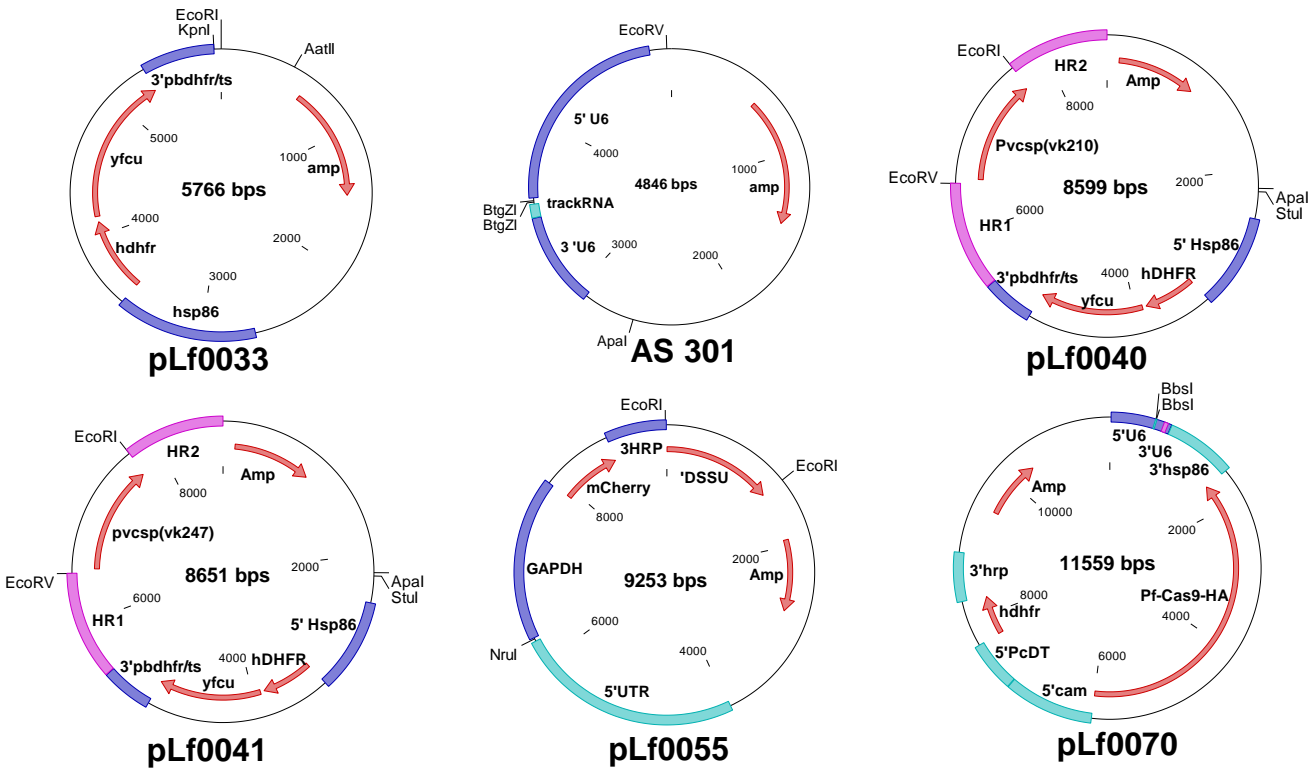

Cas9/sgRNA-Cas9 expression plasmids

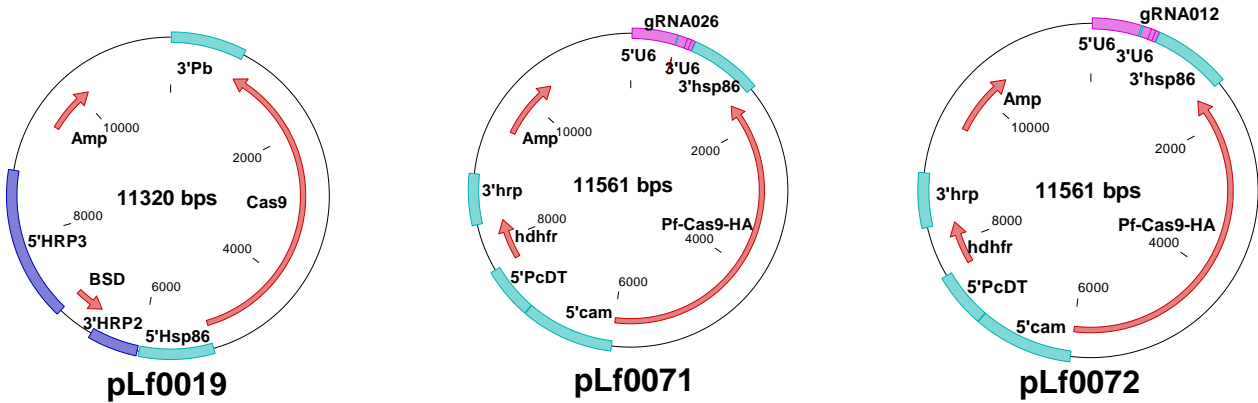

sgRNA-donor DNA/ donor DNA plasmids

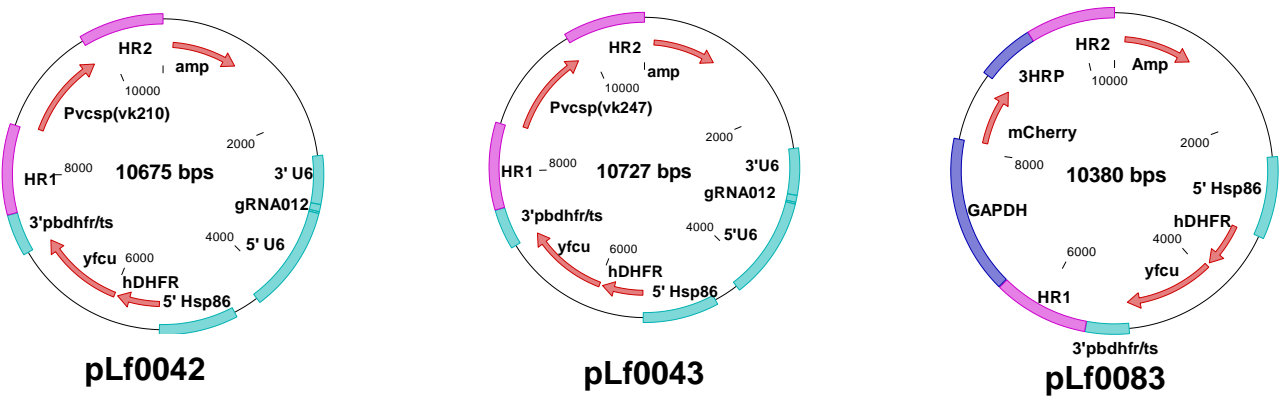

Supplement: Supplementary file 1 — Additional file 1. Vector maps of the P. vivax csp genes introduced into P. falciparum. A. Vector maps of the different plasmids used to generate two chimeric P. falciparum parasite lines (pf-pvcsp) expressing P. vivax CSP(VK210) of CSP(VK247) and a P. falciparum line lacking expression of CSP (PfΔcsp). See “Methods” section for description and details of the generation of these plasmids. [file 12936_2018_2431_MOESM1_ESM.pdf]

A

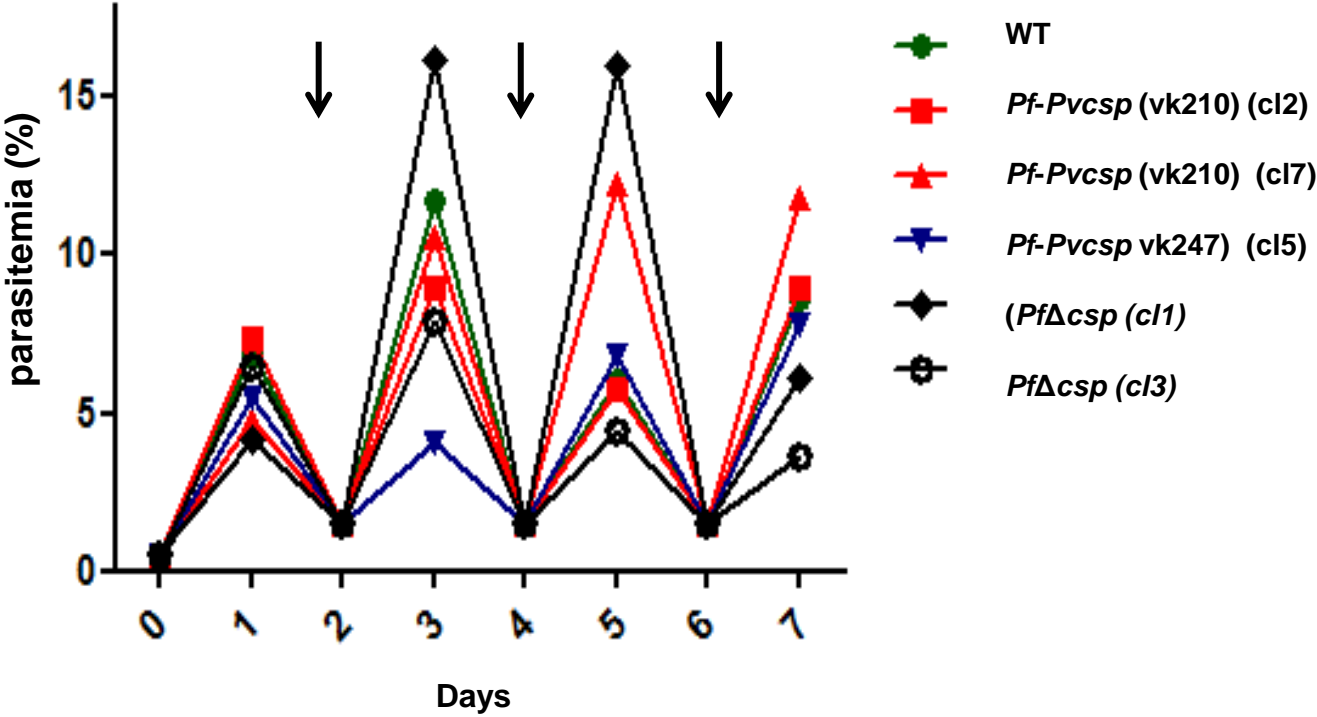

B

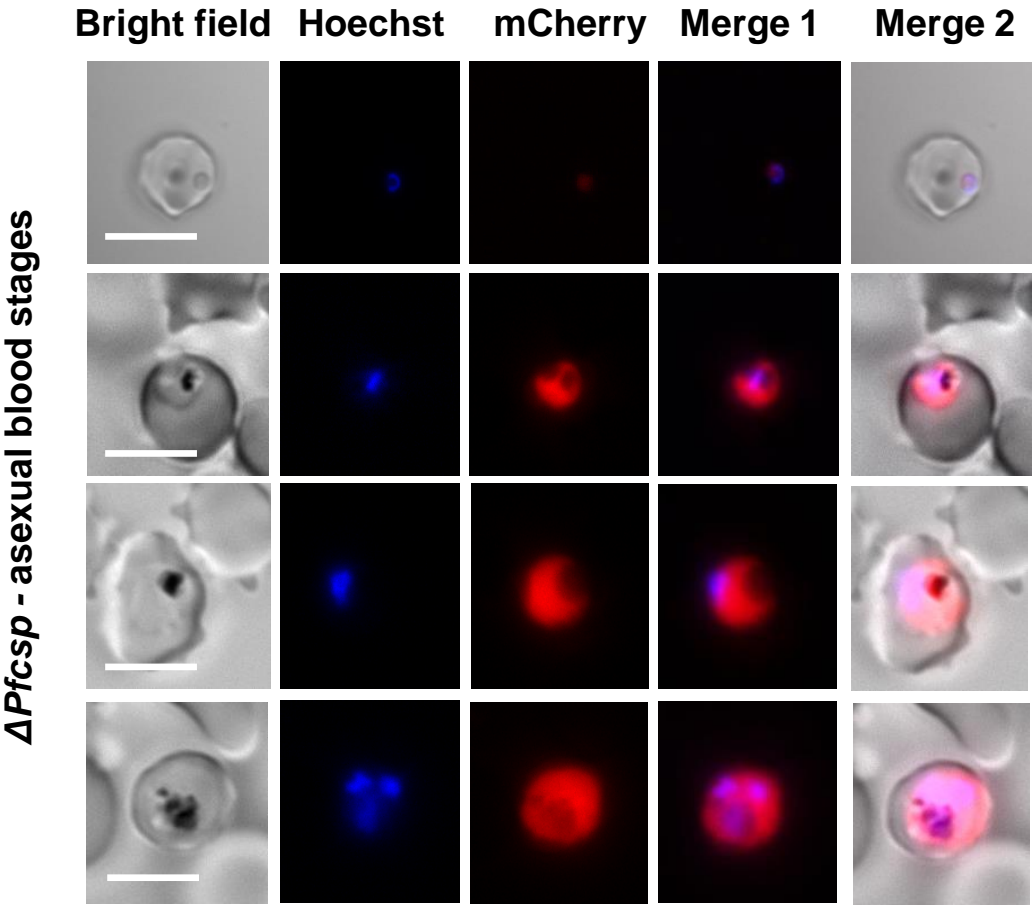

Supplement: Supplementary file 4 — Additional file 4. Growth of asexual blood stages and mCherry expression in blood stages. Growth of asexual blood stages of two chimeric P. falciparum parasite lines (pf-pvcsp(vk210) and pf-pvcsp(vk247), a P. falciparum line lacking expression of CSP (PfΔcsp) and P. falciparum wild type (WT) parasites. Parasites of the different cloned lines were cultured in semi-automated culture system for a period of 7 days. Cultures were initiated with a parasitaemia of 0.5%. Arrows indicate the dilution of the cultures with fresh red blood cells to have a final parasitaemia of 1%. B. mCherry-expressing blood stages of PfΔcsp parasites where the csp gene has been disrupted by insertion of an mCherry expression cassette (see Additional file 5 for details of the generation of PfΔcsp). Scale bar, 7 µm. [file 12936_2018_2431_MOESM4_ESM.pdf]

*Pf-Pvcsp*  
(vk210)

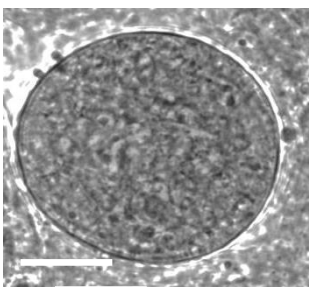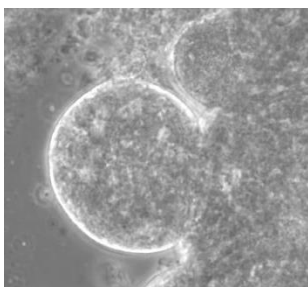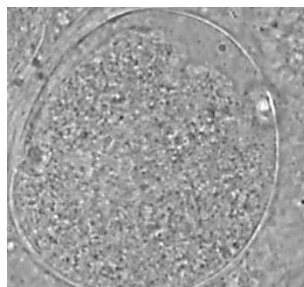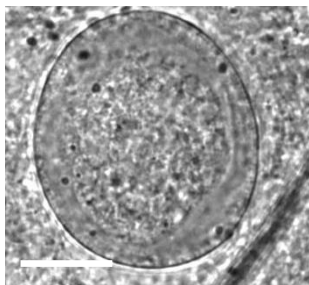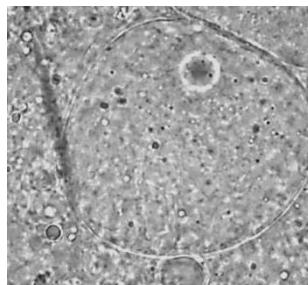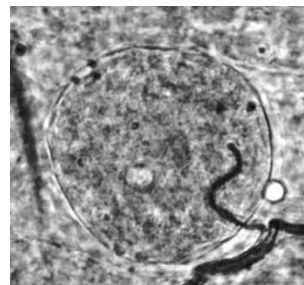

*Pf-Pvcsp*  
(vk247)

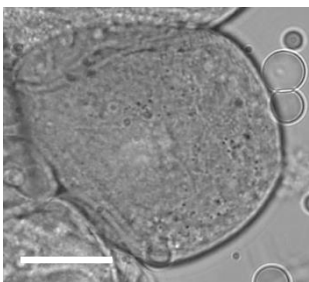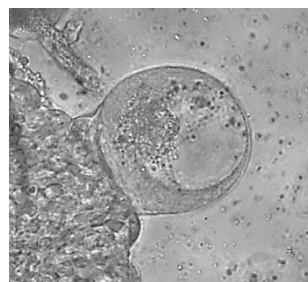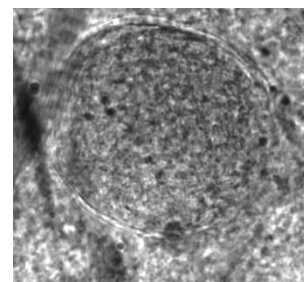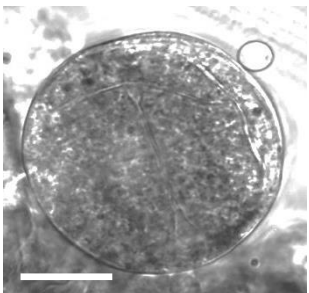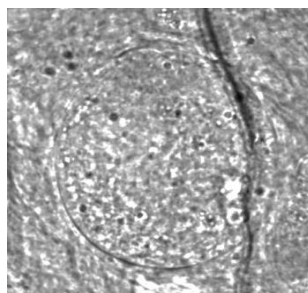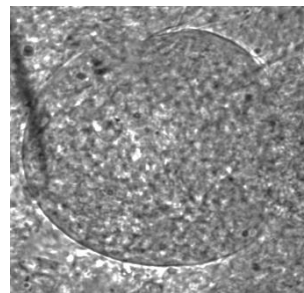

*pf* $\Delta$ CSP

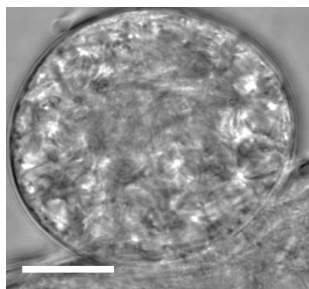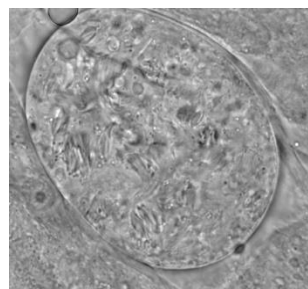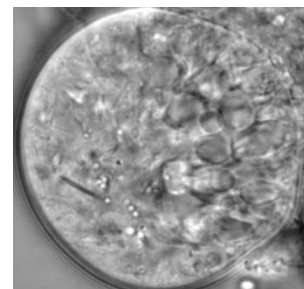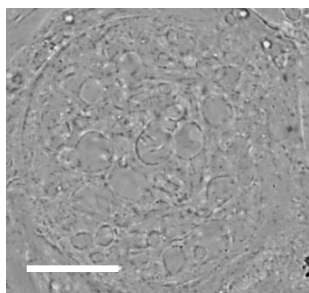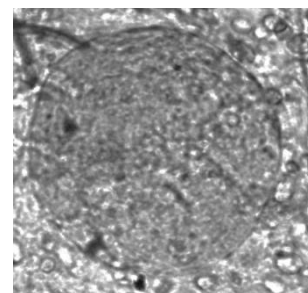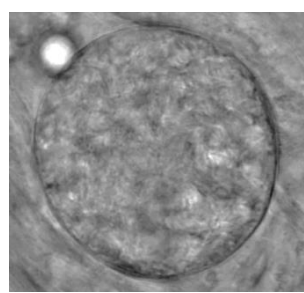

Supplement: Supplementary file 5 — Additional file 5. Degenerated oocysts of two chimeric P. falciparum parasite lines (pf-pvcsp) and a PfCSP knockout line (PfΔcsp). Light microscope pictures of degenerated oocysts at day 10 after feeding gametocytes to Anopheles stephensi mosquitoes. These oocysts are classified as degenerate based on the absence of sporozoite formation and vacuolated cytoplasm. See Fig. 2 for pf-pvcsp and wild type P. falciparum (WT) oocyst in which sporozoite formation occurred. No sporozoite formation was observed in PfΔcsp oocysts. Scale bar, 20 µm. [file 12936_2018_2431_MOESM5_ESM.pdf]

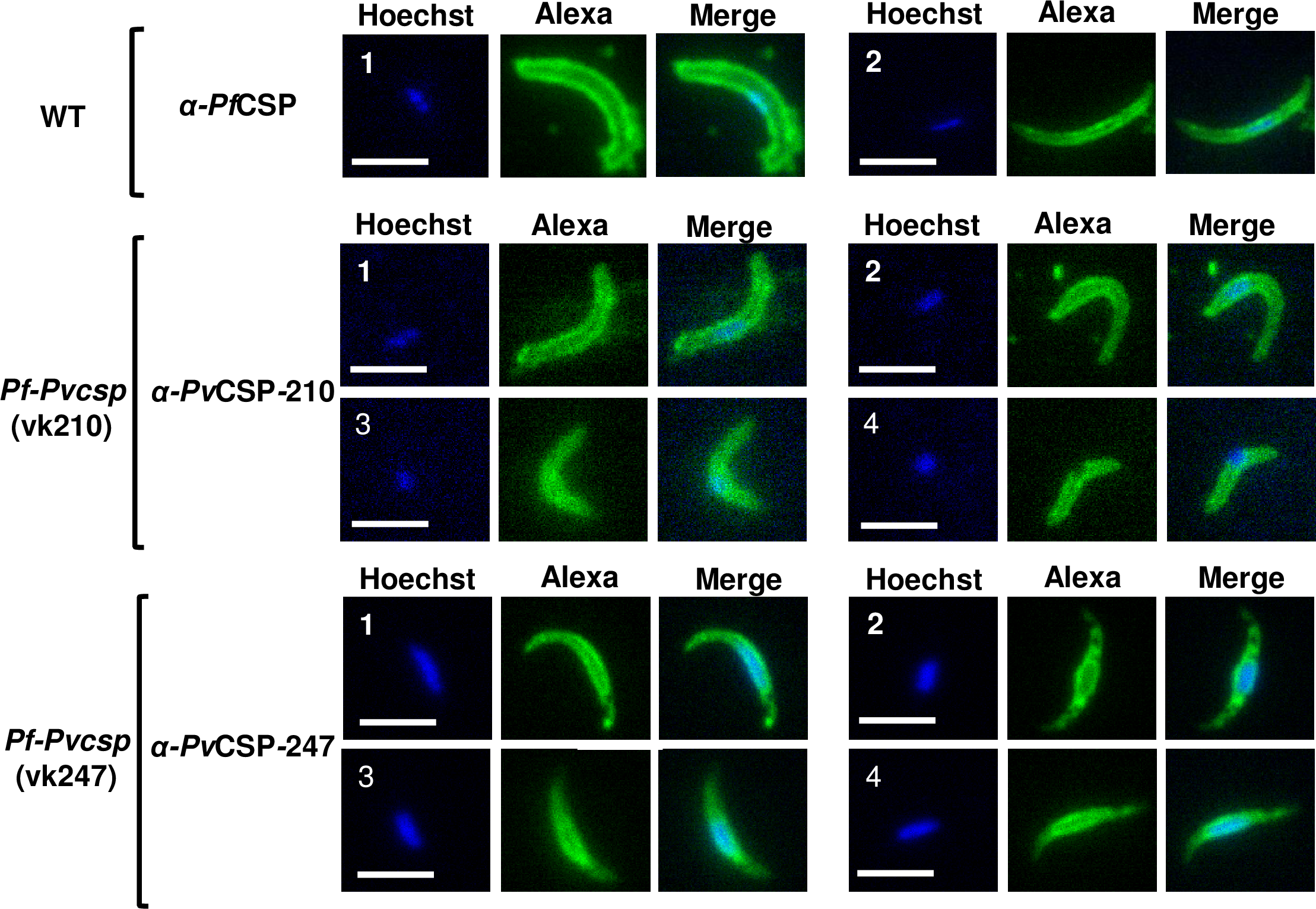

Supplement: Supplementary file 6 — Additional file 6. PvCSP(VK210) and PvCSP(VK247) expression in oocyst-derived sporozoites of two chimeric P. falciparum parasite lines (pf-pvcsp). Immunofluorescence analyses of wild type P. falciparum (WT) sporozoites and oocyst-derived pf-pvcsp sporozoites. Fixed sporozoites were labelled with mouse anti-PvCSP-VK210 mAb, anti-PvCSP-VK247mAb and mouse anti-PfCSP antibodies. Secondary conjugated antibodies used: anti-IgG Alexa Fluor® 488 (green. Nuclei stained with the DNA-specific dye Hoechst-33342. All pictures were recorded with standardized exposure/gain times; Alexa Fluor® 488 (green) 0.7 s; Hoechst (blue) 0.136 s; bright field 0.62 s (1× gain). Scale bar, 7 µm. [file 12936_2018_2431_MOESM6_ESM.tif]

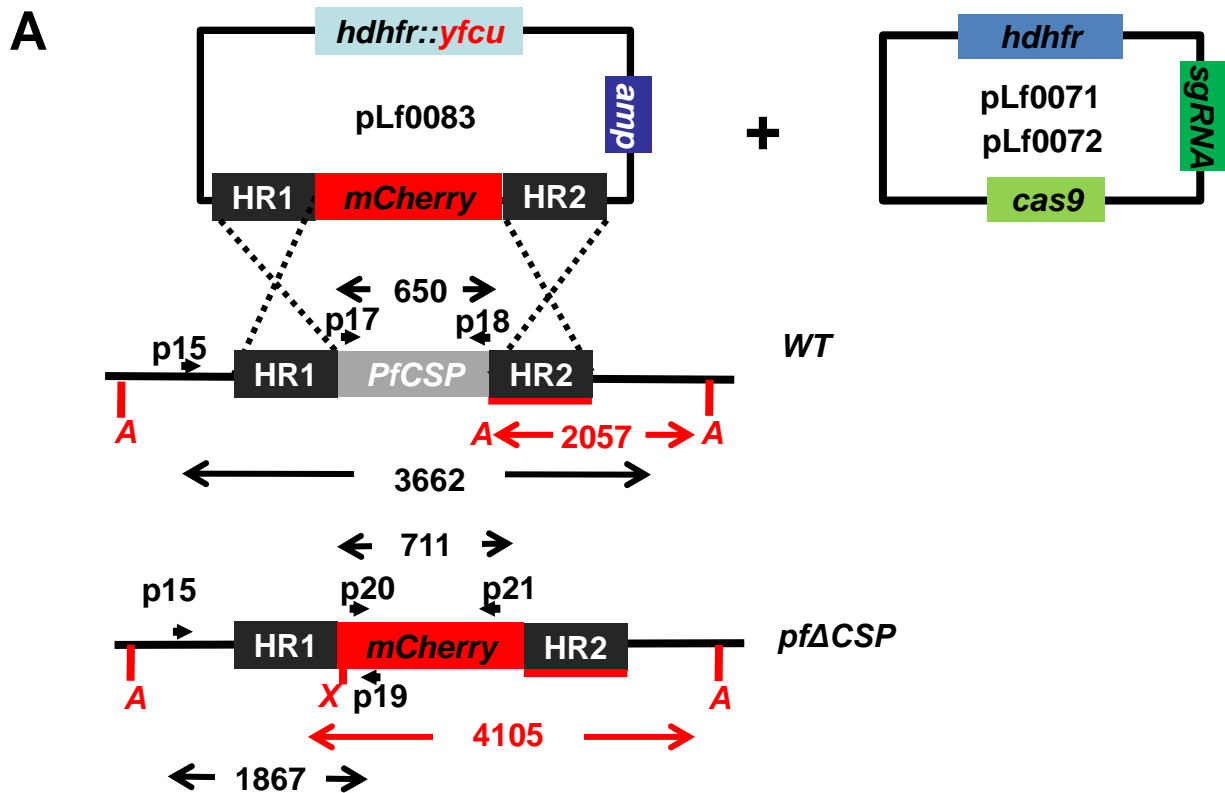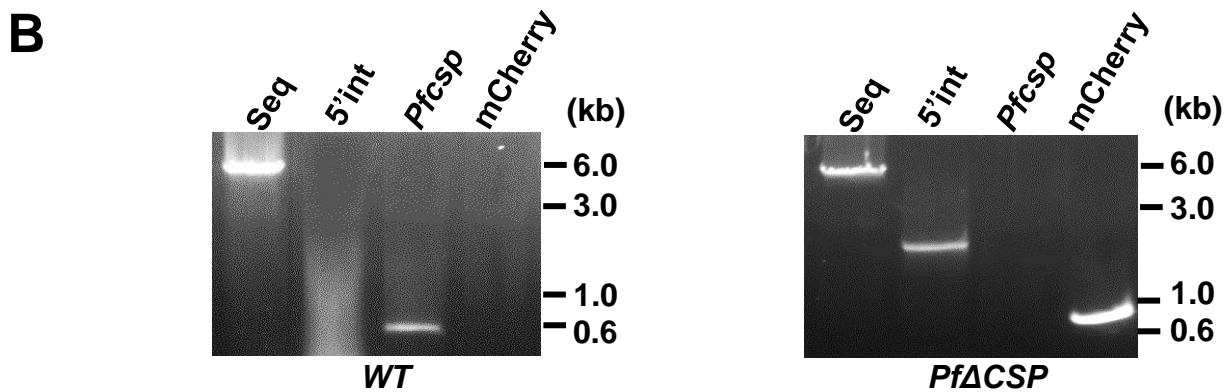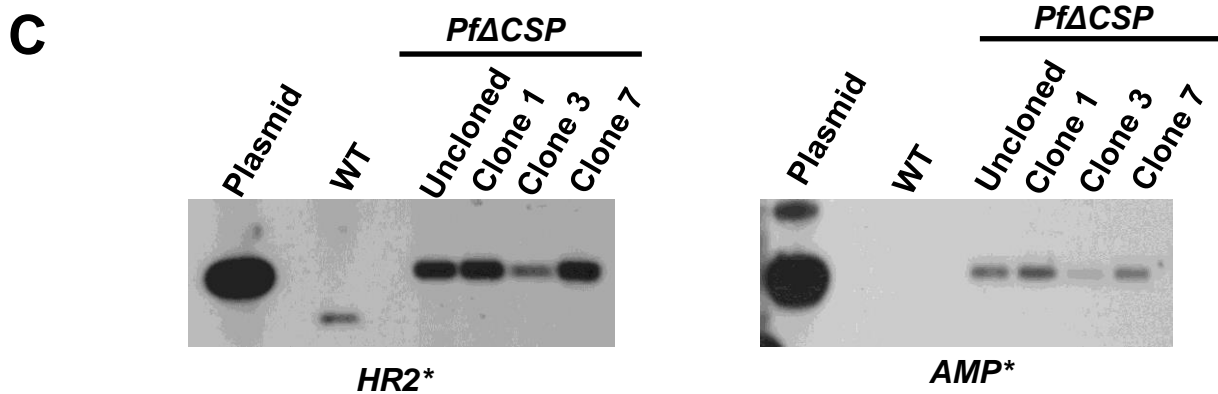

Supplement: Supplementary file 7 — Additional file 7. Generation and genotyping a P. falciparum mutant line lacking expression of CSP (PfΔcsp). A. The PfΔcsp line was generated using CRISPR/Cas9 methodology. The pfcsp gene was replaced by insertion of a mCherry expression cassette (mCherry under control of the of the constitutive gapdh promoter) using donor-DNA plasmids pLf0086. A schematic representation of the pfcsp locus before and after insertion of the construct showing the location of the restriction sites (A: AvaII), sizes (in bp) of restriction fragments (red for Southern blot analysis), location of primers (p), PCR amplicons and sizes of the fragments (in black) used to analyse correct disruption of the pfcsp and insertion of the mCherry cassette (B, C). HR1, HR2: pfcsp homology (targeting) regions. The figure is not shown to scale. Primer sequences can be found in Additional file 8. B. Diagnostic PCR confirming correct integration of the mCherry cassette into the PfCSP locus. 5′ integration PCR (lane 2; primers p15/p19); pfcsp open reading frame (lane 3; primers p17/p18); P. falciparum sequestrin as a control gene (lane 1; primers p22/p23); mCherry gene (lane 4; primers P20/P21) of cloned parasites of PfΔcsp (cl3) and WT. C. Southern blot analysis of AvaII/XhoI restricted DNA of WT and PfΔcsp parasites confirms the specific integration of the mCherry cassette into the pfcsp gene locus. DNA was hybridized with a probe targeting the homology region 2 (upper panels; HR2; primers p3/p4; see (A) of pfcsp. The hybridization pattern observed with the HR2 probe identified the expected different-sized DNA fragments in WT and pf-pvcsp parasites (2057 bp and 4105 bp). In addition to show absence of donor-DNA plasmid and presence of single cross-over events DNA was hybridized with a probe for the ampicillin gene (lower panels; intermediate donor-DNA plasmid pLf0040 digested with AatII and PvuI). [file 12936_2018_2431_MOESM7_ESM.pdf]
