# Supplementary material for: Chimeric Plasmodium falciparum parasites expressing Plasmodium vivax circumsporozoite protein fail to produce salivary gland sporozoites
Source: Malar J. 2018 Aug 9;17:288. doi: 10.1186/s12936-018-2431-1 (PMC6085629; doi:10.1186/s12936-018-2431-1)
Supplement: Supplementary file 2 — Additional file 2. List of primers used in this study. [file 12936_2018_2431_MOESM2_ESM.pdf]

List of primers used in this study

Primer ID	Gene ID	Sequence	Enzymes	Product (bp)	Description
P1	PF3D7_0304600	ATAGGTACCAGCACGTGATAAAGTAATTG	*Kpn*I	1007	Forward HR 1 *pfcs*
P2	PF3D7_0304600	ACGGAATTCCGGCGGATATCTGTAATTTATAATATACGTGG	*EcoR*I/*EcoR*V		Reverse HR 1 pfcs
P3	PF3D7_0304600	CATAAGAATTCAGAACACATCTTAGTTTGAG	*EcoR*I		Forward HR 2 *pfcs*
P4	PF3D7_0304600	AATCGACGTCTTAGCTTTTAGTATAGGATAG	*Aat*II	927	Forward HR 2 *pfcs*
P5		GGGGATATCGCGGCCGCACAAAATCTATATATACACGCATATATTTAAAATG	*EcoR*V/*Not*I	1188	Forward ORF pvcsp-vk210/247
P6		AGAATGAATTCTCATGCGTAATGTTTATTTAATTAAATAATGC	*EcoR*I		Reverse ORF pvcsp-vk210/247
P7	PF3D7_0304600	TAAGTATATAATATTTAAGGCCTCAACAAATAAAAGTTTTAGAGCTAGAA			Forward gRNA012
P8	PF3D7_0304600	TTCTAGCTCTAAAACTTTTATTTGTTGAGGCCTTAAATATTATATACTTA			Reverse gRNA012
P9		CATTTGGATTTCTACACATCTTG			Sequencing gRNA012
P10		TAGGAAATAATAAAAAAGCACC			Sequencing gRNA012
P11	PF3D7_0304600	TATTATGGAAGTTCGTCAAACACA	*Bbs*I		Forward gRNA026
P12	PF3D7_0304600	AAACTGTGTTTGACGAACTTCCAT	*Bbs*I		Reverse gRNA026
P13	PF3D7_0304600	TATTTAAGGCCTCAACAAATAAAA			Forward gRNA012 *Pf csp ko*
P14	PF3D7_0304600	AAACTTTTATTTGTTGAGGCCTTA			Reverse gRNA012 *Pf csp ko*
P15	PF3D7_0304600	GCTTATAGTCATATACCTAATACG		3676,3728 (*Pf-Pvcsp),* 3662 (WT)	Forward long range PCR
P16	PF3D7_0304600	GAACACCGTATGATTATATGAC			Reverse long range PCR
P17		GATGGAAATAACGAAGACAACGAG		650	Forward *Pf csp* orf
P18		CATCTACATTTCGGTTTGGGTCA			Reverse *Pf csp* orf
P19		CCAATTATTGCTGATTTATACAAATG		With P15 1867	Reverse 5'integration
P20		AAAGGTACCTAAAAGAAATATGAGAAC		711	Forward mCherry
P21		AAAAAGCTTTTTCGCCACAGGAGAAAC			Reverse mCherry
P22	PF3D7_0405300	AACGCTAGCTTCCGGATCGCTGTCTTTAC		5400	Forward Sequestrin
P23	PF3D7_0405300	AGCCGCGGCATGGAGAAGGGTTCTATTTTATCG			Reverse Sequestrin
P24		CGCGTAATACGACTCACTATAGGGC			LR-PCR sequencing
P25		CAGTGTGATGGATATCTGCAG			LR-PCR sequencing
P26		CATGCAGATATAAAAAGGTAGAAG			LR-PCR sequencing
P27		AGCAGAACCTAAAAATCCAAG			LR-PCR sequencing
P28		CATTTCCACCAGCTGCTTG			LR-PCR sequencing
P29		CCGGGGTACCAGTTGTGTAACATAAATGTTTCTC			LR-PCR sequencing
P30		CATGTGGTGTTGGAGTTAGAG			LR-PCR sequencing
P31		TTTATGCTTCCGGCTCGTATG			LR-PCR sequencing
P32		ggggatatcgcggccgcACAAAATCTATATATACACGCATATATTTAAAATG			LR-PCR sequencing
