# Supplementary material for: Chimeric Plasmodium falciparum parasites expressing Plasmodium vivax circumsporozoite protein fail to produce salivary gland sporozoites
Source: Malar J. 2018 Aug 9;17:288. doi: 10.1186/s12936-018-2431-1 (PMC6085629; doi:10.1186/s12936-018-2431-1)

A

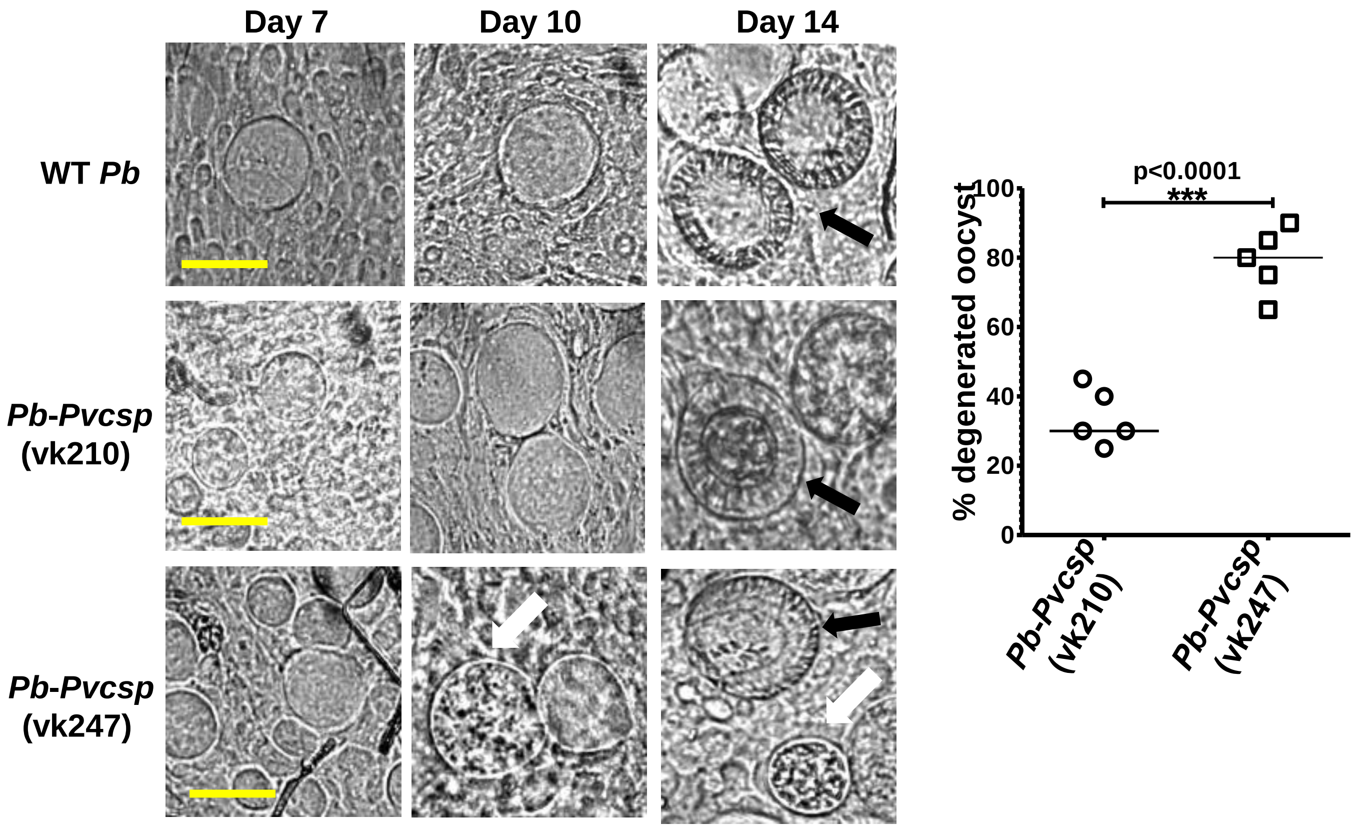

Parasite line	Oocyst no. (mean ± D)	Sporozoite no. (mean ± SD)	Significant different from WT (Student t-test)
WT Pb	116 ± 22	22,800 ± 3453	NA
***Pb-Pvcsp*(vk210)**	112 ± 37	18,700 ± 3755	p = 0.11 (NS)
***Pb-Pvcsp*(vk247)**	101 ± 28	4,200 ± 1549	p < 0.0001 (****)

B

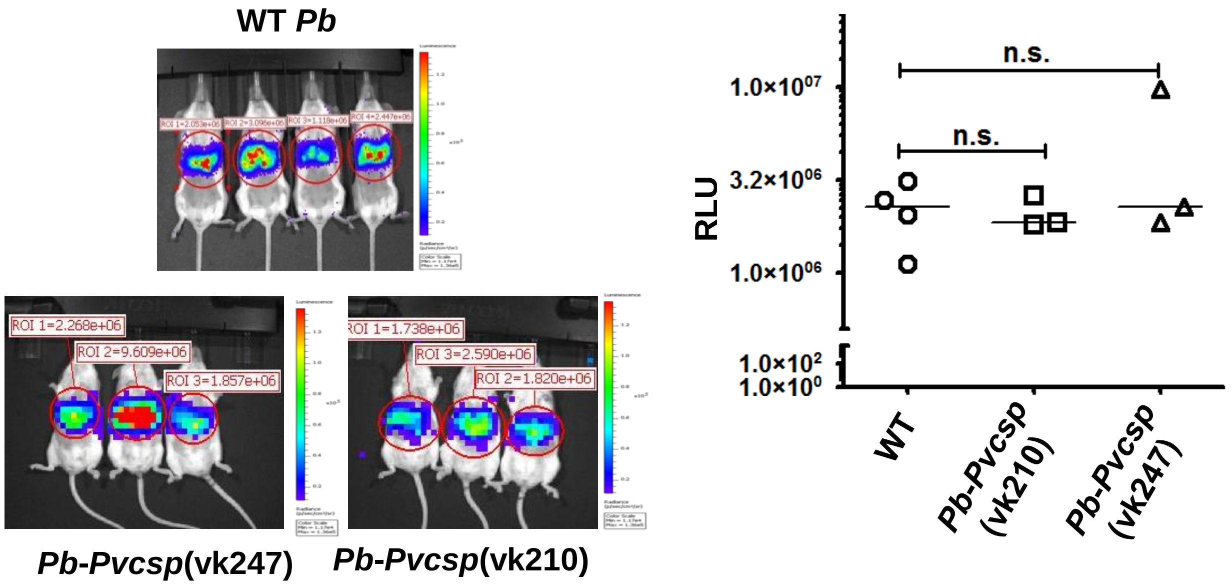

Supplement: Supplementary file 8 — Additional file 8. Chimeric rodent parasite lines pb-pvcsp(vk210) and pb-pvcsp(vk247) are able to produce salivary gland sporozoites that are infectious to mice. A. Upper panel, left: Light microscope pictures of wild type P. berghei (WT) and pb-pvcsp oocyst at days 7, 10 and 14 after feeding of An. stephensi mosquitoes. Black arrows indicate oocyst with sporozoite formation and white arrows indicate degenerated, vacuolated oocyst without sporozoite formation (scale bar, 10 µm). Upper panel, right, Percentage of degenerated oocyst in An. stephensi mosquitoes (n = 5) at day 14 after feeding (***P = < 0.0001; unpaired T-test). Lower panel: Oocyst and sporozoite production of wild type P. berghei (WT) and pb-pvcsp parasites. B. In vivo imaging of parasite liver loads at 44 h after injection of mice with salivary gland sporozoites of wild type P. berghei (WT) and pb-pvcsp parasites. Left panel: luminescence signals in the different groups of mice. Right panel: quantification of the bioluminescence signals in the different groups of mice measured as Relative Luminescence Units (RLU). [file 12936_2018_2431_MOESM8_ESM.pdf]
