# Supplementary material for: Chimeric Plasmodium falciparum parasites expressing Plasmodium vivax circumsporozoite protein fail to produce salivary gland sporozoites
Source: Malar J. 2018 Aug 9;17:288. doi: 10.1186/s12936-018-2431-1 (PMC6085629; doi:10.1186/s12936-018-2431-1)
Supplement: Supplementary file 3 — Additional file 3. Sequence analysis of the P. vivax csp genes introduced into P. falciparum. Long-range PCR fragments (see Fig. 1) were cloned into pET TOPO TA (Invitrogen) and sequenced. See Additional file 2 for primer sequences used for sequencing the complete fragment. A. LR-PCR fragment of parasites of pf-pvcsp(vk210). B. LR-PCR fragment of parasites of pf-pvcsp(vk247). [file 12936_2018_2431_MOESM3_ESM.pdf]

A

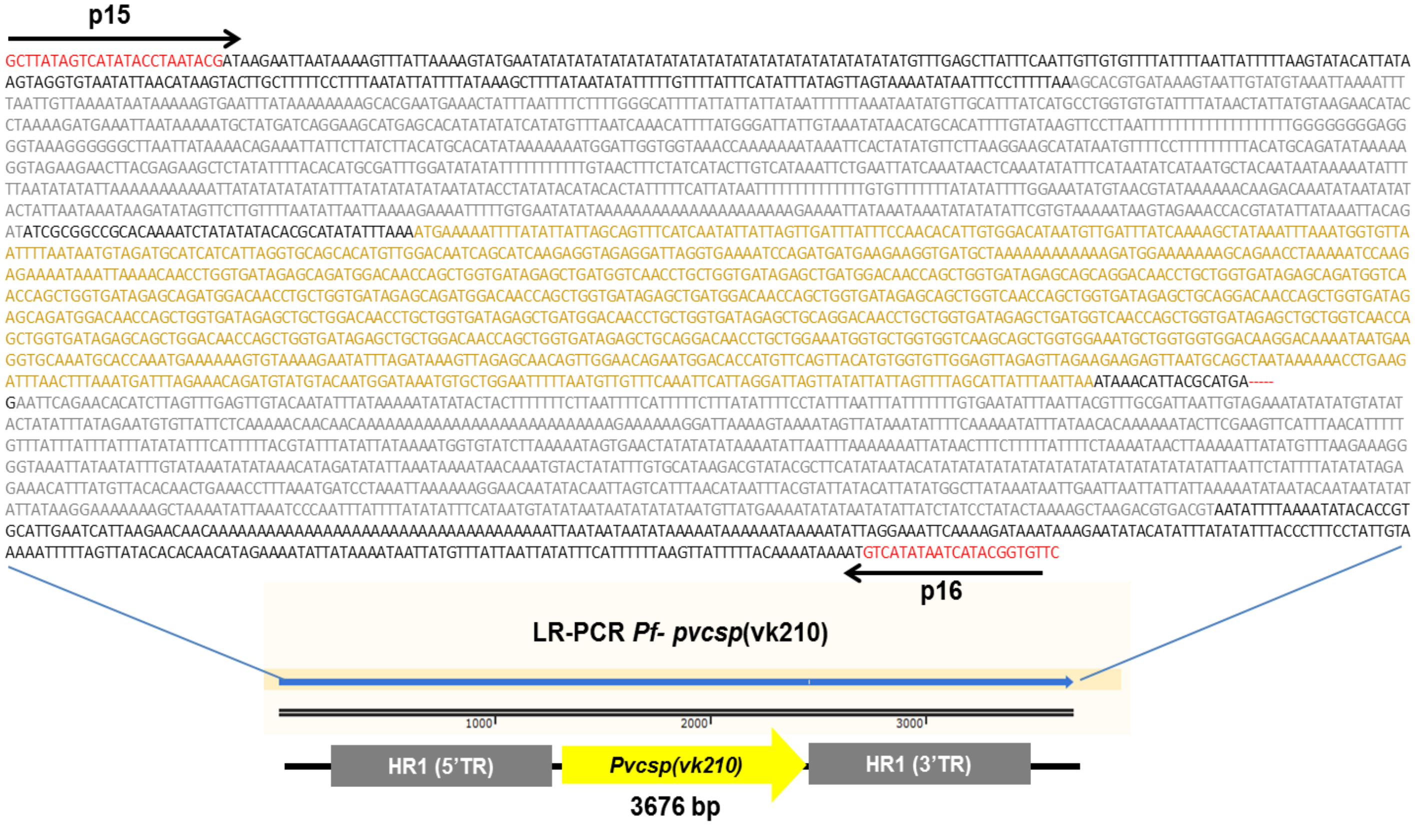
p15
p16
LR-PCR Pf- pvcsp(vk210)
1000
2000
3000
HR1 (5'TR)
Pvcsp(vk210)
HR1 (3'TR)
3676 bp

B

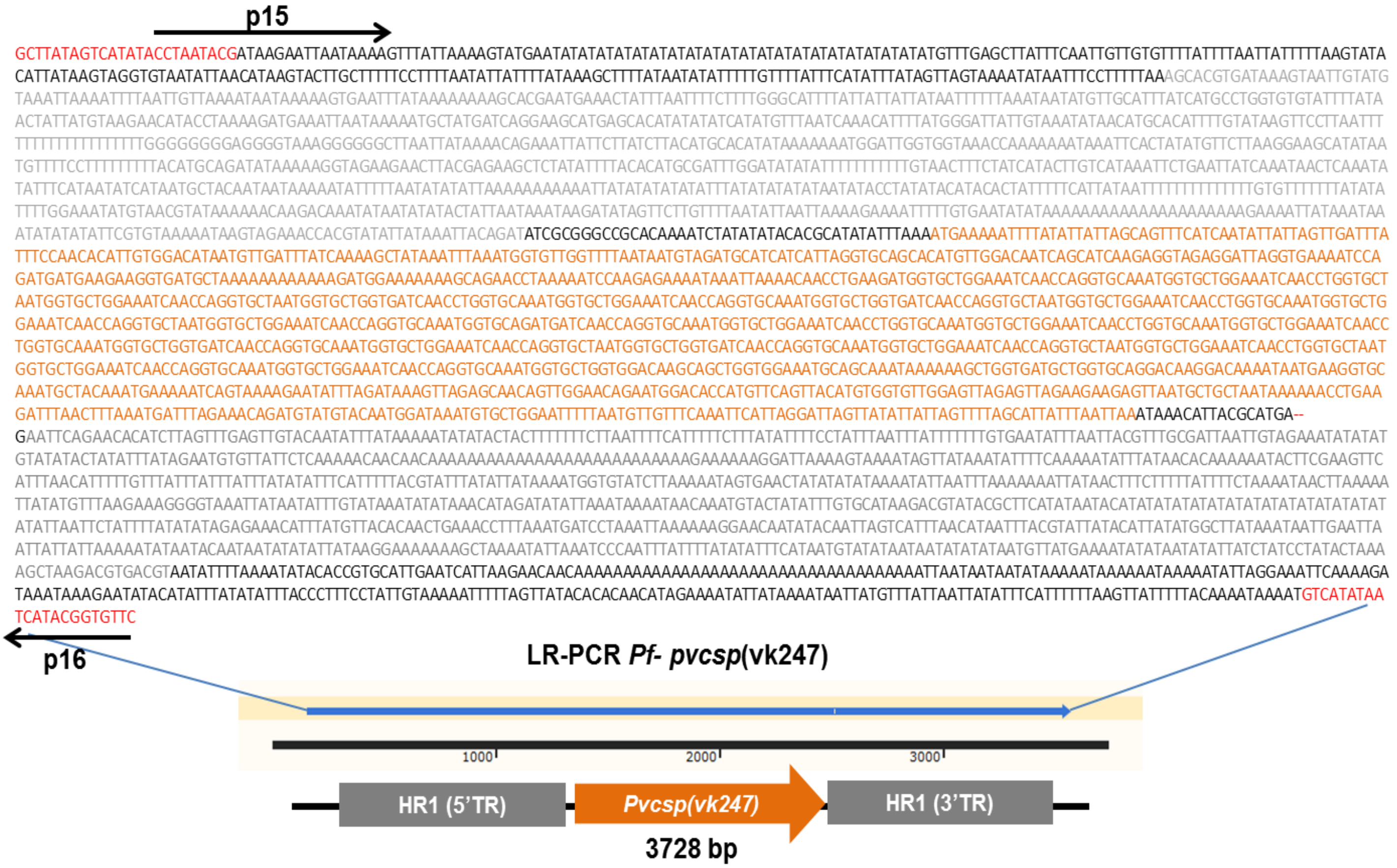
p15
p16
LR-PCR Pf- pvcsp(vk247)
1000
2000
3000
HR1 (5'TR)
Pvcsp(vk247)
HR1 (3'TR)
3728 bp
